# Intestinal peroxisomal fatty acid β-oxidation regulates neural serotonin signaling through a feedback mechanism

**DOI:** 10.1371/journal.pbio.3000242

**Published:** 2019-12-05

**Authors:** Aude D. Bouagnon, Lin Lin, Shubhi Srivastava, Chung-Chih Liu, Oishika Panda, Frank C. Schroeder, Supriya Srinivasan, Kaveh Ashrafi

**Affiliations:** 1 Department of Physiology, University of California San Francisco, San Francisco, California, United States of America; 2 Dorris Neuroscience Center, The Scripps Research Institute, La Jolla, California, United States of America; 3 Boyce Thompson Institute, Cornell University, Ithaca, New York, United States of America; UMass Med School, UNITED STATES

## Abstract

The ability to coordinate behavioral responses with metabolic status is fundamental to the maintenance of energy homeostasis. In numerous species including *Caenorhabditis elegans* and mammals, neural serotonin signaling regulates a range of food-related behaviors. However, the mechanisms that integrate metabolic information with serotonergic circuits are poorly characterized. Here, we identify metabolic, molecular, and cellular components of a circuit that links peripheral metabolic state to serotonin-regulated behaviors in *C*. *elegans*. We find that blocking the entry of fatty acyl coenzyme As (CoAs) into peroxisomal β-oxidation in the intestine blunts the effects of neural serotonin signaling on feeding and egg-laying behaviors. Comparative genomics and metabolomics revealed that interfering with intestinal peroxisomal β-oxidation results in a modest global transcriptional change but significant changes to the metabolome, including a large number of changes in ascaroside and phospholipid species, some of which affect feeding behavior. We also identify body cavity neurons and an *ether-a-go-go* (EAG)–related potassium channel that functions in these neurons as key cellular components of the circuitry linking peripheral metabolic signals to regulation of neural serotonin signaling. These data raise the possibility that the effects of serotonin on satiety may have their origins in feedback, homeostatic metabolic responses from the periphery.

## Abstract 2

As a neuromodulator, serotonin regulates numerous behavioral and emotional states. This study shows in the nematode C. elegans, metabolic cues that originate in the organism's periphery modulate neurally regulated serotonergic processes, including feeding behavior and egg laying, revealing a homeostatic signal that informs the nervous system of the organism's nutritional state.

## Introduction

In both invertebrate and vertebrate species, behaviors such as feeding, movement, reproduction, and learning are influenced by nutritional and metabolic signals [1–3]. In mammals, the nervous system actively monitors internal nutritional status by directly sensing specific metabolites like carbohydrates, amino acids, and fatty acids, in addition to sensing endocrine signals derived from peripheral tissues [4,5]. These internal nutrient cues are integrated with environmental stimuli and past experiences to orchestrate cohesive and context-appropriate behavioral and physiological responses. Defects in internal metabolic sensing processes contribute to the development of a number of disorders, including diabetes, obesity, impaired immune function, neurodegeneration, and accelerated aging [3,6–8]. Thus, elucidating the mechanisms by which nutrient status is sensed and communicated between tissues is of critical importance in understanding metabolic homeostasis as well as how metabolism influences myriad physiological and pathophysiological conditions.

Like mammals, *C*. *elegans* display a range of behavioral and physiological responses to changes in nutrient availability [1,9]. Moreover, as in vertebrate species, the neuromodulator 5-hydroxytryptamine, 5-HT, commonly referred to as serotonin, is a key mechanism through which information about food availability is converted to behavioral, physiological, and metabolic responses in *C*. *elegans* [10–12]. For example, even in the presence of food, worms that lack serotonin display the feeding, egg-laying, movement, and metabolic rates that are normally seen when wild-type animals are deprived of food [13]. In contrast, pharmacologic or genetic manipulations that elevate serotonin signaling elicit the range of responses seen when plentiful food supplies are present [14,15]. Importantly, serotonin signaling is not simply an on/off indicator of food availability but the extent of serotonin signaling allows for animals to fine-tune their responses based on their nutritional status and past experiences [16–18]. One illustration of this is the effects of varying levels of serotonin signaling on pharyngeal pumping rate, the mechanism by which *C*. *elegans* ingest nutrients [19]. *C*. *elegans* that have been moved off of their *Escherichia coli* food source exhibit reduced serotonin signaling and reduced pumping rates. Both serotonin signaling and pumping rates are elevated as animals are returned to food [20,21]. However, if animals experience a period of fasting before they are returned to food, they exhibit an even further elevation in feeding rate compared to animals that have only been off of food for a brief period of time. The hyper-elevated feeding behavior is accounted for by correspondingly elevated secretion of serotonin from specific neurons [17]. The hyper-secretion of serotonin and the corresponding hyper-elevated feeding are transient and animals eventually resume the intermediate levels of serotonin signaling and feeding rates seen in well-fed animals [17,21]. Thus, the low, high, and intermediate levels of serotonin signaling correspond to the low, high, and intermediate pharyngeal pumping rates, respectively.

In addition to modulating food intake behavior, serotonin signaling also affects energy metabolism [22]. *C*. *elegans* that have been returned to food after a period of fasting transition from a metabolic state that favors energy conservation to an active state of energy utilization. This active metabolic state is driven by elevated serotonin signaling [12,23,24]. If elevated levels of serotonin are maintained by pharmacological or genetic interventions, *C*. *elegans* exhibit fat loss [15]. The effects of serotonin on body fat are not simply a by-product of its effects on food intake, as we and other groups have found that molecular and cellular circuits that link serotonin signaling to peripheral energy metabolism are largely independent from those that regulate feeding [12,14,15,23,24]. For example, serotonin secreted from the ADF sensory neurons signals through neurally expressed SER-5 serotonergic receptor to modulate feeding. Yet the SER-5 receptor is not required for the serotonergic regulation of peripheral fat metabolism [12,21]. Instead, serotonin signals through the MOD-1 receptor on URX neurons to promote the release of a neuroendocrine signal that activates triglyceride lipolysis and fatty acid oxidation [12,23,24].

In a prior study, we discovered that specific components of lipid oxidation pathways can elicit regulatory effects on feeding behavior [12]. Here, we build upon that finding and describe a feedback mechanism that links peripheral energy metabolism to neuronal serotonin signaling. We find that loss of ACOX-1, a peripheral acyl–coenzyme A (CoA) oxidase that catalyzes a key step in peroxisomal fatty acid oxidation, affects feeding and egg-laying responses, two behaviors regulated by serotonin. Blunting the utilization of fatty acyl-CoA species, the metabolic substrates of ACOX-1, results in rewiring of peripheral metabolic pathways. Lastly, we find that the URX body cavity neuron serves as a cellular conduit linking peripheral metabolic status to neural serotonin signaling.

## Results

### *acox-1* mutants are unresponsive to the feeding stimulatory effects of serotonin

**Fig 1:**
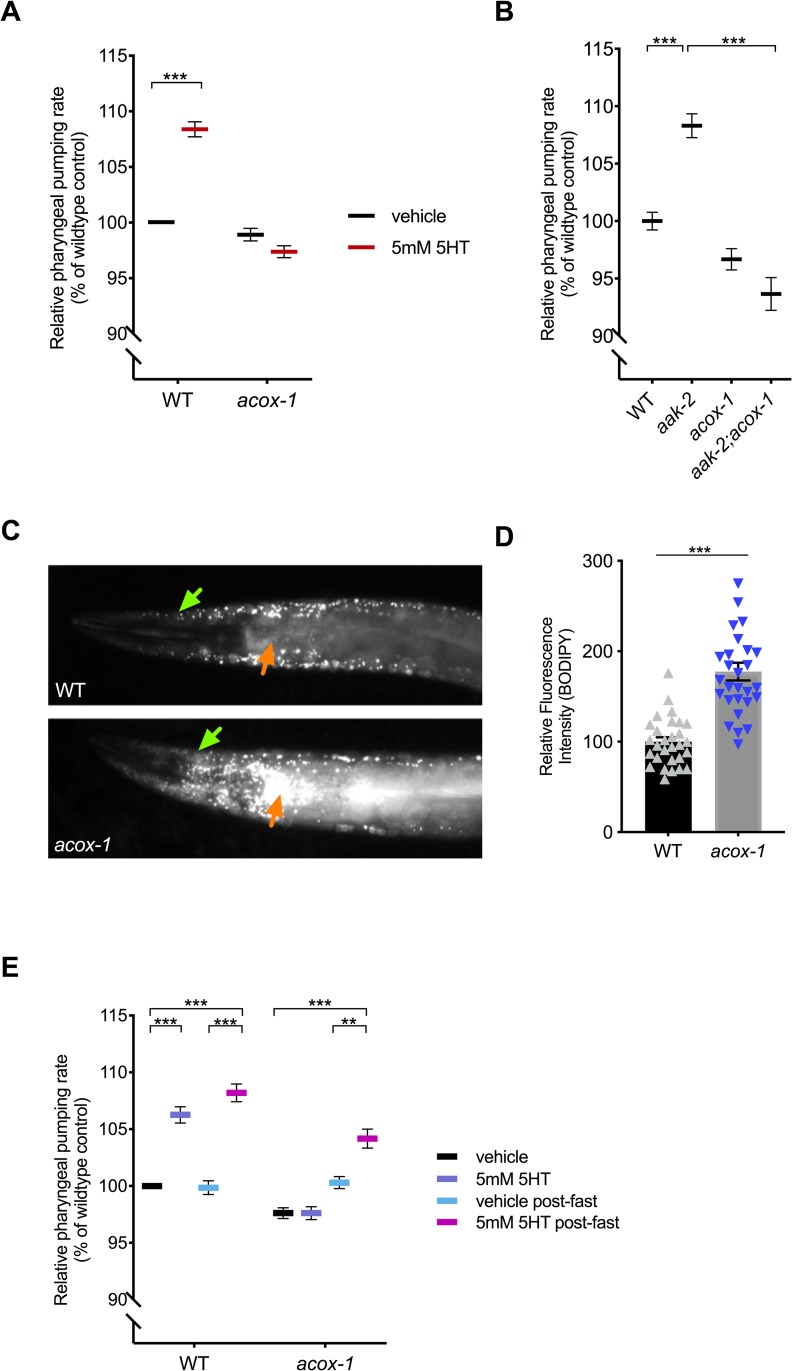
*acox-1* mutants are insensitive to the feeding stimulatory effects of serotonin. **(A)** *acox-1(ok2257)* mutants are resistant to pharyngeal pumping increasing effects of 5 mM serotonin. **(B)** Loss of *acox-1* suppresses the elevated feeding rates of *aak-2* animals. All feeding data are normalized to vehicle-treated wild-type animals and are presented as a percentage of wild-type rates. In both **(A)** and **(B)**, error bars indicate ±SEM from normalized mean, *n* = 50 animals per strain. ***p < 0.0001 ANOVA (Tukey). **(C** and **D)** *acox-1* mutants accumulate significantly more fat than wild-type animals as assessed by epidermal (green arrows) and intestinal (orange arrows) BODIPY fluorescence levels. Representative images of BODIPY staining **(C)** and corresponding quantifications of epidermal BODIPY fluorescence levels **(D)** ***p < 0.001, Student *t* test. **(E)** Fasting *acox-1* mutants for 90 minutes restores their ability to elevate feeding in response to 5 mM serotonin. Day 1 adult animals were either fed ad libitum or fasted for 90 minutes, then plated on vehicle or 5 mM 5HT plates for 60 minutes before assessing pharyngeal pumping rates. Error bars indicate ±SEM from normalized mean, *n* = 15 animals per condition. **p < 0.01, ***p < 0.001 ANOVA (Tukey). See S1 Table and S1 Data for underlying data. *aak-2*, AMP-activated kinase alpha 2; *acox-1*, acyl-coenzyme A oxidase 1; BODIPY, boron dipyrromethene; WT, wild-type; 5HT, 5-hydroxytryptamine.

While the serotonergic regulation of fat metabolism is largely distinct from serotonergic regulation of feeding, we previously noted two exceptions. RNA interference (RNAi)–mediated inactivation of either *acox-1*, encoding a peroxisomal acyl-CoA oxidase, or *cpt-6*, encoding a mitochondrial carnitine palmityoltransferase, blocked both the fat-reducing and the feeding-increasing effects of serotonin [12]. Interestingly, *acox-1* and *cpt-6* function as entry points to peroxisomal and mitochondrial β-oxidation pathways, respectively [25]. To further investigate how metabolic pathways affect serotonin signaling, we focused on *acox-1* given the availability of a null mutant for this gene at the time that the study was undertaken. Recapitulating our prior RNAi findings, ad libitum fed *acox-1(ok2257)* animals exhibit wild-type feeding rates but are unresponsive to the feeding stimulatory effects of exogenous serotonin (Fig 1A, S1A Fig). Similar results were obtained whether pumping rates were measured manually for short, 10-second intervals or minute-long video recordings (S1B Fig). We previously demonstrated that elevated levels of serotonin signaling exert their effects on fat and feeding pathways by inactivating AMP-activated kinase (AMPK) complexes in distinct neurons [15,21]. As in mammals, the catalytic subunit of AMPK can be encoded by one of two distinct genes, *aak-1* and *aak-2*, in *C*. *elegans* [26]. Elevated levels of serotonin signaling inactivate the AAK-2 subunit, and the hyperactive pumping rate of serotonin-treated wild-type animals is recapitulated by *aak-2* mutants [21]. Loss of *acox-1* suppresses the elevated feeding rates of *aak-2* mutants, suggesting that the effects of *acox-1* on feeding are not restricted to exogenously supplied serotonin (Fig 1B).

Although the above findings suggested that *acox-1* functions downstream or parallel to serotonin signaling, we sought to rule out the possibility that *acox-1* affects serotonin biosynthesis. Transcription of *tph-1*, the gene that encodes tryptophan hydroxylase, the rate-limiting enzyme of de novo serotonin synthesis, is highly dynamic and can be modulated by a range of external and internal cues including food availability, food quality, and stress [27,28]. We observed no changes in the transcriptional expression of *tph-1* or in direct quantifications of serotonin levels in *acox-1* mutants (S1C and S1D Fig). In mammals, defects in peroxisomal fatty acid oxidation pathways are associated with neurodevelopmental disorders due to toxic accumulation of long and very-long chain fatty acids [29]. We had previously shown that elevation of serotonin signaling from the chemosensory, amphid ADF neurons is sufficient to cause elevated pharyngeal pumping [21]. We therefore considered the possibility that the lack of response to serotonin in *acox-1* mutants may be the indirect consequence of a defect in the ADF neurons or other sensory neurons. As one broad examination of neural morphology and development, we used DiI dye staining and found that *acox-1* mutants had properly structured amphid sensory neurons (S1E Fig). Collectively, we found no evidence that the inability of serotonin to elevate feeding rate in *acox-1* mutants is due to deficiencies in serotonin biosynthesis or structural abnormalities in serotonergic sensory neurons.

Based on homology to mammalian acyl-CoA oxidase 1, ACOX-1 is predicted to catalyze the first and rate-limiting step in peroxisomal β-oxidation [30,31]. We used fluorescence intensity of BODIPY labeled fatty acids as a measure of fat accumulation because we and others have previously demonstrated that BODIPY fluorescence corresponds to biochemical and label-free methods, such as Coherent anti-Stokes Raman Scattering spectroscopy, measurements of fat content [32]. We found that animals lacking ACOX-1 accumulate significantly more fat in their skin-like epidermis and intestines, the two major sites of fat metabolism, consistent with the notion that *acox-1* mutants have a reduced capacity to break down lipids (Fig 1C and 1D). If the inability of *acox-1* mutants to increase their feeding rates represented a homeostatic response to elevated internal energy stores, we predicted that a period of nutrient depletion should reverse *acox-1* mutants’ feeding behavior. We fasted *acox-1* mutants for 90 minutes, a period of time shown to elicit a coordinated shift in internal metabolic networks towards fat mobilization and energy production, and reintroduced fasted or ad libitum fed animals to either vehicle- or serotonin-containing plates [33,34]. Fasting restored the ability of *acox-1* mutants to increase their feeding rates in response to exogenous serotonin, suggesting that *acox-1* mutants do not simply have a generalized defect in pharyngeal pumping and that a period of starvation can reverse the feeding regulatory effect induced by loss of ACOX-1 activity (Fig 1E). Together, these findings suggest that loss of ACOX-1 activity leads to the generation of a homeostatically regulated signal that can rapidly and reversibly modulate serotoninergic feeding circuits.

### Intestinal ACOX-1 regulates feeding behavior

**Fig 2:**
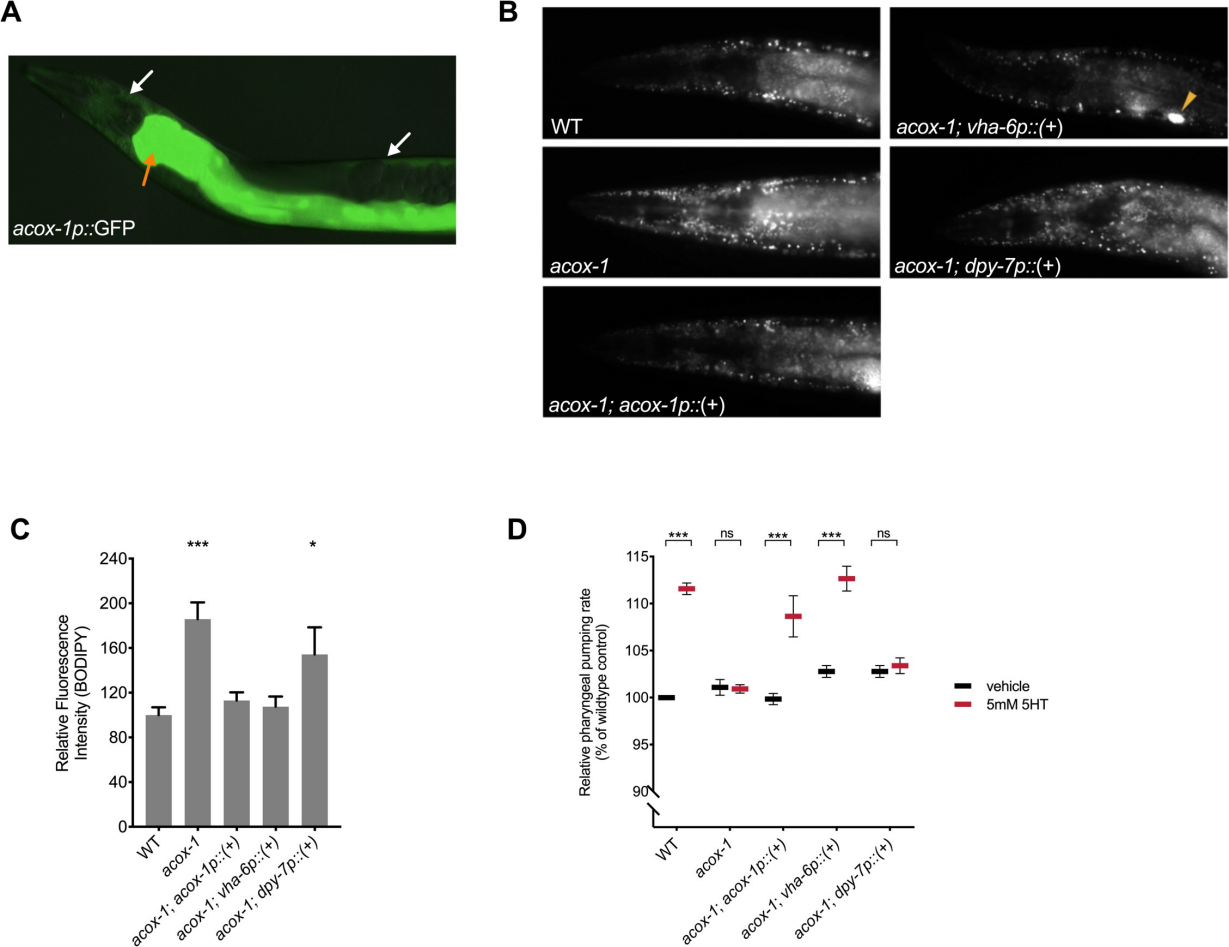
Reconstitution of *acox-1* in the intestine rescues fat and feeding phenotypes. **(A)** *acox-1* is expressed in epidermal (white arrows) and intestinal (orange arrow) tissues. Merged DIC and green epifluorescent image of a transgenic animal expressing an *acox-1p*::*gfp* transcriptional reporter. **(B** and **C)** Reconstitution of *acox-1* gDNA under its own promoter (*acox-1p*) and under an intestine-specific promoter (*vha-6p*), but not under an epidermal-specific promoter (*dpy-7p*), normalizes BODIPY fat levels. Representative images of BODIPY staining **(B)** and corresponding quantifications **(C)** expressed as fluorescence intensity relative to wild-type animals. The yellow arrow in **(B)** indicates a coelomocyte co-injection marker used during transgenic strain generation. Error bars indicate ±SEM from normalized mean, *n* > 20 per strain. *p < 0.05, ***p < 0.001, ANOVA (Tukey). **(D)** Reconstitution of *acox-1* gDNA under an intestine-specific promoter (*vha-6p*) restores serotonin responsiveness. Feeding data are normalized to vehicle-treated wild-type animals and are presented as a percentage of wild-type rates. Error bars indicate ±SEM from normalized mean, *n* > 15 per strain. ***p < 0.001, ANOVA (Tukey). See S1 Table and S1 Data for underlying data. *acox-1*, acyl-coenzyme A oxidase 1; BODIPY, boron dipyrromethene; DIC, differential interference contrast; gDNA, genomic DNA; ns, not significant; WT, wild-type; 5HT, 5-hydroxytryptamine.

To elucidate the site of ACOX-1 activity in regulating feeding behavior, we performed tissue-specific rescue experiments. As previously reported, ACOX-1 is expressed in the epidermis and the intestine [30] (Fig 2A). Expression of a full-length wild-type *acox-1* genomic DNA (gDNA) sequence in the intestine (via the *vha-6* promoter) but not in the epidermis (via the *dpy-7* promoter) normalized fat levels in *acox-1* mutants (Fig 2B and 2C). The intestine is a major metabolic organ in *C*. *elegans* and carries out numerous functions, including food digestion, nutrient absorption, and packaging and secretion of lipids [35]. We found that the intestinal expression of wild-type *acox-1* restored the capacity of *acox-1* mutants to elevate their feeding rates in response to serotonin treatment (Fig 2D). Thus, intestinal ACOX-1 activity can affect neuronal serotonergic feeding circuits.

### Modulation of feeding by ACOX-1 requires fatty acyl-CoA synthesis

**Fig 3:**
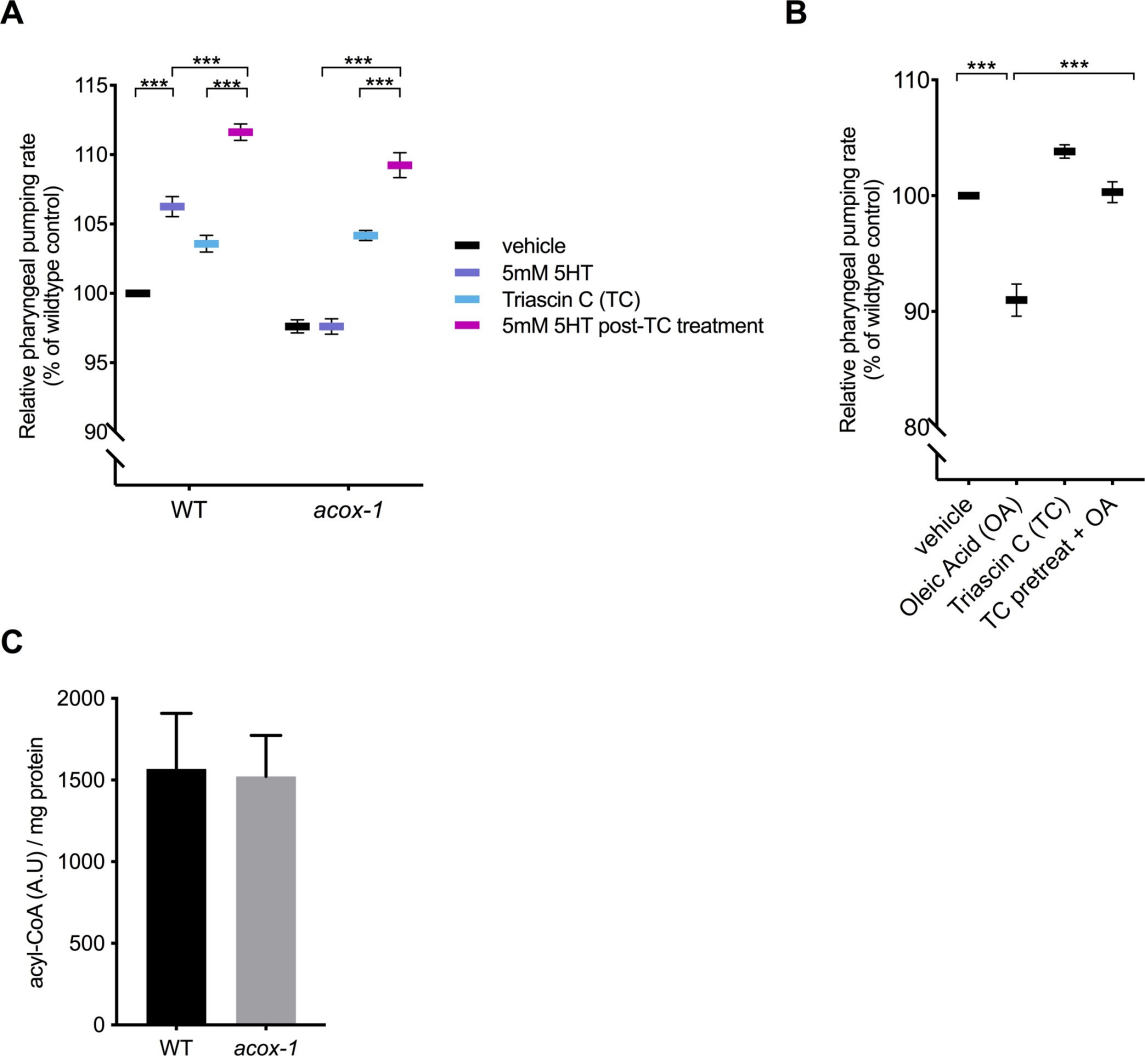
Modulation of feeding by ACOX-1 requires fatty acyl-CoA synthesis. **(A)** Inhibiting ACSs in *acox-1(ok2257)* animals restores the ability to elevate feeding in response to 5 mM serotonin. Day 1 adult animals were pretreated with vehicle or 1 μM ACS inhibitor Triacsin C for 90 minutes before being plated on vehicle or 5 mM 5HT plates. Feeding rates were assessed after 60 minutes on assay plates. Feeding data are normalized to vehicle-treated wild-type animals and are presented as a percentage of wild-type rates. Error bars indicate ±SEM from normalized mean, *n* = 15 animals per condition. ***p < 0.001 ANOVA (Tukey). **(B)** Animals were treated with 1 mM oleic acid (OA) or 1 μM Triacsin C (TC) for one hour, or pretreated with 1 μM TC for one hour prior to OA treatment before feeding was assayed. Error bars indicate ±SEM from normalized mean, *n* > 15 per strain. ***p < 0.001, ANOVA (Tukey). See S1 Table and S1 Data for underlying data. At the concentration used (0.0002%), DMSO had no effect on pumping rate. **(C)** Acyl-CoA levels in *acox-1* mutants are unchanged relative to WT. Acyl-CoA species were separated and quantified by HPLC and normalized to protein concentrations, *n* = 3 independent extractions. ACOX-1, acyl-coenzyme A oxidase 1; ACS, acyl-coenzyme A synthetase; CoA, co-enzyme A; HPLC, high-pressure liquid chromatography; WT, wild-type; 5HT, 5-hydroxytryptamine.

Acyl-CoA oxidases regulate the rate of metabolic flux through peroxisomes as they govern the first and rate-limiting reaction in peroxisomal metabolic pathways, specifically catalyzing the desaturation of long and very-long chain fatty acyl-CoA esters to 2-trans-enoyl-CoAs [36]. The *C*. *elegans* genome encodes seven acyl-CoA oxidases that form homo- and heterodimer complexes with distinct substrate specificities [37]. Structural and biochemical analyses suggest that ACOX-1 homodimers are capable of accommodating a wide range of fatty acyl-CoA substrates and contribute to peroxisomal β-oxidation of ascaroside lipids [38,39]. Given the enzymatic function of ACOX-1, we hypothesized that its loss leads to an accumulation of fatty acyl-CoA species, leading to anorectic effect. Fatty acyl-CoAs are generated by acyl-CoA synthetases (ACSs), a family of enzymes that esterify free fatty acids with co-enzyme A (CoA) [40]. To prevent the formation of acyl-CoA thioesters, we acutely exposed animals to Triacsin C, an inhibitor of ACS activity [41]. This treatment restored the ability of serotonin to cause an elevated feeding rate in *acox-1* mutants (Fig 3A). Furthermore, Triacsin C also rescued feeding defects in *acox-1;aak-2* mutants (S2A Fig). There are at least 20 known or predicted ACSs encoded in the *C*. *elegans* genome. As a strategy to validate the Triacsin C results and identify the specific synthetases involved, we screened through 18 of 20 *acs* genes for which RNAi clones were available. RNAi treatment against multiple *acs* genes, most notably those of *acs-18*, *acs-20*, or *acs-22*, suppressed the feeding defects of *acox-1* mutants, suggesting a degree of redundancy among *C*. *elegans* ACSs (S2B Fig). As we did not establish the efficacy of each of the 18 RNAis, we cannot rule out the possibility that other ACS enzymes also contribute to acyl-CoA pools used by ACOX-1. To further test the notion that fatty acyl-CoAs modulate feeding, we treated animals with oleic acid, a dietary fatty acid. This led to a reduction in pharyngeal pumping rate (Fig 3B). The oleic acid–induced feeding suppression was abrogated in animals that were pretreated with Triacsin C, suggesting that the anorectic effect is induced by oleoyl-CoA or a downstream metabolic derivative.

To directly evaluate whether loss of *acox-1* causes accumulation of acyl-CoAs, we employed a high-pressure liquid chromatography (HPLC)–based extraction and detection method from whole animal extracts [42,43]. We found that acyl-CoAs levels were not grossly altered in *acox-1* mutants (Fig 3C). One possible explanation for the absence of elevated acyl-CoA species in *acox-1* mutants is that only a small fraction of total acyl-CoAs in the animals are directed to peroxisomal β-oxidation, such that a change in their abundance may not be detectable by our assay. Moreover, acyl-CoA metabolism is known to be highly spatially regulated and acyl-CoA products are selectively synthesized or partitioned in specific tissues [44]. Yet another possibility is that acyl-CoAs are substrates for numerous metabolic processes and can be converted into a variety of signaling molecules like ceramides, ascarosides, and eicosanoids [45,46]. Thus, blocking acyl-CoA utilization by inactivating ACOX-1 may shunt these intermediates into a variety of other metabolic derivatives that ultimately elicit anorectic effect.

### Loss of *acox-1* results in modest transcriptional up-regulation of compensatory fat oxidation pathways

To better understand molecular responses to the loss of *acox-1*, we compared the transcriptome of *acox-1* mutants to that of wild-type animals using RNA-sequencing. Loss of *acox-1* had a surprisingly limited effect on global gene expression. Our analyses revealed that only 36 out of approximately 17,000 tested genes were differentially expressed in *acox-1* mutants, with only three genes significantly up-regulated. Among the differentially expressed genes, the majority were expressed in the intestine, consistent with our finding that this tissue is a major site of action for ACOX-1. Gene Ontology analysis revealed an enrichment for genes involved in “lipid metabolic” and “innate immune” related processes (Table 1). Among the differentially regulated genes, several are predicted to encode for components of peroxisomal β-oxidation, including a homolog of acyl-CoA oxidase (ACOX-2), an enoyl-CoA hydratase (ECH-1.1), and an ortholog of human bile acid-CoA:amino acid N-acyltransferase (K05B2.4). Two lipases, LIPL-2 and K03H6.2, whose activities are predicted to promote lipid mobilization, were down-regulated. Collectively, we interpret these results to mean that the transcriptional responses elicited upon loss of *acox-1* likely compensate for peroxisomal dysfunction by limiting lipid mobilization and by up-regulating alternative lipid utilization pathways. Using RNAi, we asked whether inactivation of any of the up-regulated genes could counteract the resistance of *acox-1* mutants to serotonin-induced feeding elevation, but the experiment yielded no such candidates.

**Table 1:** Loss of acox-1 results in modest transcriptional changes in intestinal and fatty acid metabolic pathways.

WormBase ID	Gene Name	log_2_(FC)	Cellular Process	Putative Function	*p*-value	Adjusted *p*-value
**UP-REGULATED**	*** ***	** **		** **	** **	** **
WBGene00008681	scrm-4**	2.50	membrane phospholipids	phospholipid scramblase	7.83 × 10^−11^	1.48 × 10^−6^
WBGene00019404	*K05B2*.4**	1.98	peroxisomal fatty acid metabolism	ortholog of human bile acid-CoA: amino acid N-acyltransferase (BAAT)	1.18 × 10^−4^	8.71 × 10^−2^
WBGene00013540	*Y75B8A*.*3*	1.89	fatty acid metabolism	carboxylic ester hydrolase type B	1.83 × 10^−4^	1.15 × 10^−1^
**DOWN-REGULATED**	* *					
WBGene00011487	*T05E12*.6**	−1.64	unknown	unknown	5.88 × 10^−5^	5.83 × 10^−2^
WBGene00019619	asp-14**	−1.97	innate immune response	aspartic endopeptidase	3.93 × 10^−5^	5.54 × 10^−2^
WBGene00007875	dod-24**	−2.09	innate immune response	unknown	2.33 × 10^−5^	3.98 × 10^−2^
WBGene00009429	*irg-5*	−2.10	innate immune response	unknown	4.51 × 10^−5^	5.54 × 10^−2^
WBGene00009773	lipl-2**	−2.15	fatty acid metabolism	ortholog of human triglyceride Lipase F	2.67 × 10^−4^	1.36 × 10^−1^
WBGene00012671	*Y39B6A*.*9*	−2.17	unknown	unknown	2.24 × 10^−4^	1.17 × 10^−1^
WBGene00019368	*K03H6*.2**	−2.18	fatty acid metabolism	putative lipase	2.18 × 10^−4^	1.17 × 10^−1^
WBGene00001150	*ech-1*.1**	−2.18	fatty acid metabolism	enoyl-CoA hydratase, 3-hydroxyacyl-CoA dehydrogenase activity	9.90 × 10^−5^	8.24 × 10^−2^
WBGene00014562	*Y17D7B*.*7*	−2.24	unknown	unknown	4.67 × 10^−5^	5.54 × 10^−2^
WBGene00019495	*sdz-24*	−2.28	larval development	SKN-1 dependent zygotic transcript	1.96 × 10^−4^	1.15 × 10^−1^
WBGene00007331	*pho-11*	−2.29	metabolic process	ortholog of human acid phosphatase 2	7.81 × 10^−6^	1.47 × 10^−2^
WBGene00020560	*T19C3*.*2*	−2.29	unknown	unknown	1.25 × 10^−4^	8.75 × 10^−2^
WBGene00007916	*C34C6*.*3*	−2.30	unknown	unknown, contains an EGF-like domain	1.90 × 10^−4^	1.15 × 10^−1^
WBGene00006636	tsp-10**	−2.30	unknown	putative tetraspanin	5.29 × 10^−5^	5.54 × 10^−2^
WBGene00194708	*Y36E3A*.*2*	−2.31	membrane biology	unknown	1.20 × 10^−4^	8.71 × 10^−2^
WBGene00008584	*irg-4*	−2.32	innate immune response	unknown	2.23 × 10^−6^	7.00 × 10^−3^
WBGene00000747	*col-174*	−2.43	structural protein/collagen	collagen	1.04 × 10^−4^	8.24 × 10^−2^
WBGene00021337	*Y34F4*.2**	−2.47	membrane biology	putative tight-junction/claudin	7.44 × 10^−5^	7.01 × 10^−2^
WBGene00008565	acox-2**	−2.48	peroxisomal fatty acid metabolism	acyl-CoA oxidase	2.64 × 10^−6^	7.11 × 10^−3^
WBGene00009904	*F49E12*.*12*	−2.51	GPI anchor maturation	PGAP2 ortholog	5.07 × 10^−5^	5.54 × 10^−2^
WBGene00022375	*Y94H6A*.*2*	−2.56	unknown	unknown	2.22 × 10^−4^	1.17 × 10^−1^
WBGene00008905	*F17B5*.*1*	−2.61	redox biology	putative thioredoxin	1.47 × 10^−4^	9.53 × 10^−2^
WBGene00022730	*ZK402*.*3*	−2.64	unknown	unknown, contains a SPK domain	4.80 × 10^−5^	5.54 × 10^−2^
WBGene00020083	*R57*.2**	−2.65	unknown	unknown	1.44 × 10^−4^	9.53 × 10^−2^
WBGene00011665	*T09F5*.*1*	−2.66	protein glycosylation	putative galactosyltransferase	9.29 × 10^−5^	8.24 × 10^−2^
WBGene00008564	acox-1**	−2.74	peroxisomal fatty acid metabolism	acyl-CoA oxidase	2.01 × 10^−9^	1.26 × 10^−5^
WBGene00008698	*F11D11*.3**	−2.76	innate immune response	putative transmembrane glycoprotein	2.03 × 10^−4^	1.16 × 10^−1^
WBGene00022731	*ZK402*.*5*	−2.86	unknown	unknown, contains a SPK domain	1.05 × 10^−4^	8.24 × 10^−2^
WBGene00022156	*Y71G12B*.18**	−2.92	unknown	unknown	6.98 × 10^−6^	1.46 × 10^−2^
WBGene00015268	*BE0003N10*.*3*	−2.93	zinc ion binding	unknown	2.78 × 10^−7^	1.31 × 10^−3^
WBGene00010102	*F55C9*.5**	−3.05	unknown	unknown	3.07 × 10^−5^	4.82 × 10^−2^
WBGene00017020	*D1014*.*7*	−3.26	unknown	unknown	1.27 × 10^−6^	4.79 × 10^−3^
WBGene00017019	*D1014*.*6*	−4.17	protein glycosylation	unknown, putative galactosyltransferase activity	2.57 × 10^−10^	2.42 × 10^−6^

**Intestinally expressed gene products.

Abbreviations: acox-1, acyl-coenzyme A oxidase 1; BAAT, bile acid-coenzyme A: amino acid N-acyltransferase; CoA, co-enzyme A; EGF, epidermal growth factor; GPI, glycosylphosphatidylinositol; PGAP2, post-glycophosphatidylinositol attachment to protein 2; SPK, serine and arginine-rich protein kinase

### Loss of *acox-1* perturbs fatty acid ethanolamide signaling

**Fig 4:**
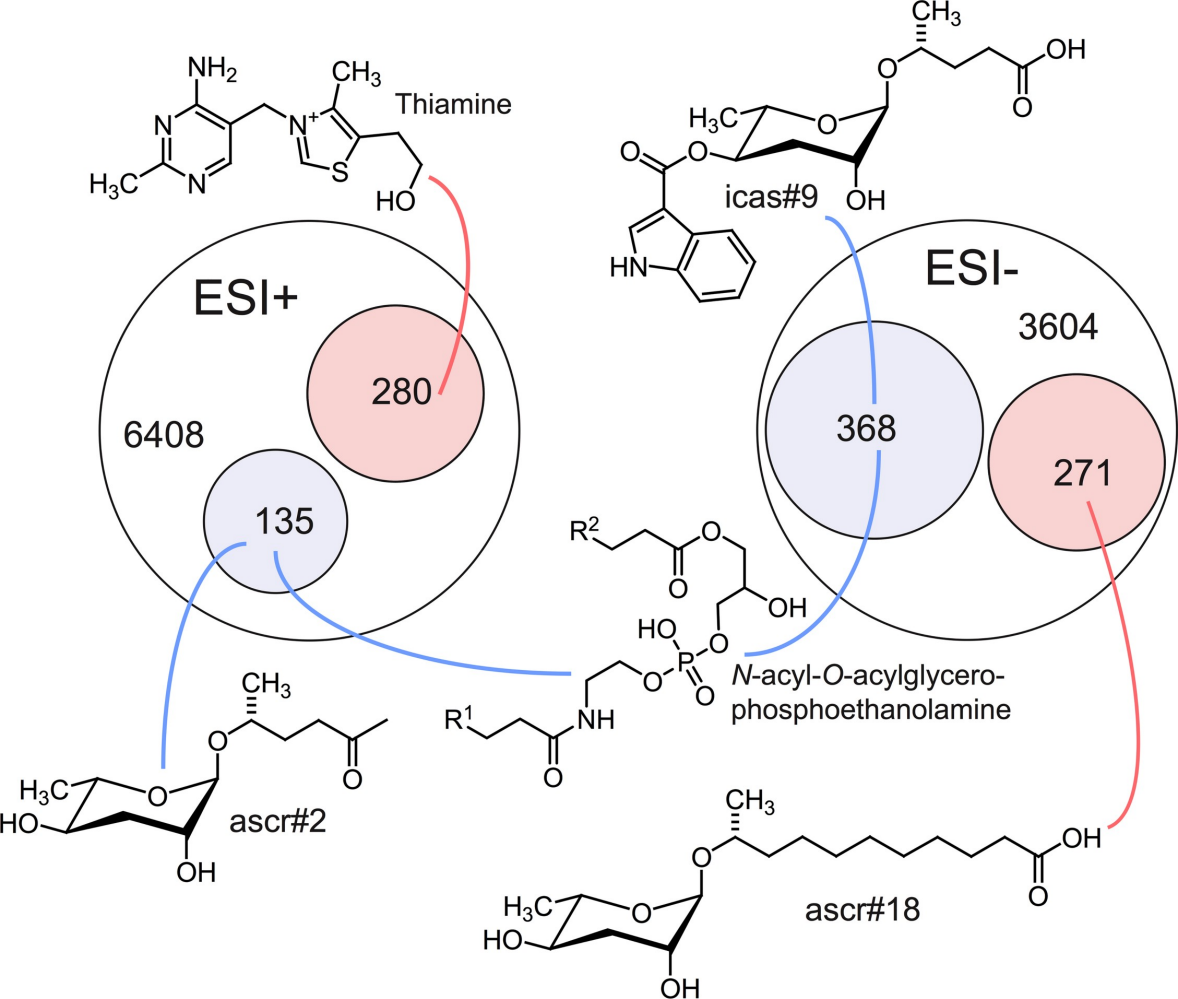
Loss of *acox-1* results in large-scale changes to the global metabolome. Venn diagrams showing total numbers of detected features (6,408 and 3,604) and numbers of metabolites more than 3-fold up-regulated (pink) or down-regulated (blue) in LC-HRMS using positive-ion (ESI+) and negative-ion (ESI−) electrospray ionization in *acox-1* mutants. Although most differentially detected features were unidentifiable, the chemical structures shown here represent examples of the most significantly up- or down-regulated compounds in identifiable metabolic classes. See S2 Table. *acox-1*, acyl-coenzyme A oxidase 1; ESI, electrospray ionization; HRMS, high-resolution mass spectrometry; LC, liquid chromatography.

Next, we sought to obtain a comprehensive overview of the impact of loss of *acox-1* on the *C*. *elegans* metabolome. For this purpose, we compared the *acox-1* mutant and wild-type metabolomes via untargeted metabolomics using HPLC–high-resolution mass spectrometry (HPLC-HRMS) and the XCMS software platform [47,48]. These analyses revealed that knockout of *acox-1* has a dramatic impact on the *C*. *elegans* metabolome. Of more than 10,000 significant features detected in the wild-type metabolome, over 500 were at least 3-fold down-regulated in *acox-1* mutants. Conversely, we detected more than 500 features that were at least 3-fold up-regulated in *acox-1* mutants. To facilitate structural classification of the vast number of detected differential features, we employed molecular networking based on analysis of tandem mass spectrometry (MS/MS) fragmentation patterns [49,50]. These analyses enabled characterization of several metabolite families up- or down-regulated in *acox-1* mutants. As expected from previous reports, we found that biosynthesis of most ascaroside pheromones with fatty acid side chains shorter than 9 carbons is abolished or strongly reduced in *acox-1* mutants, whereas abundances of ascarosides with saturated side chains of 9 to 15 carbons are 10- to 50-fold increased [38,39]. In addition, a large number of diacylglycerophosphoethanolamines (DAGPEs), primarily derived from saturated and monounsaturated fatty acids with 14 to 18 carbons, were reduced 5- to 20-fold in *acox-1* mutants. Among the metabolites most strongly up-regulated in *acox-1* mutants, thiamine and several thiamine derivatives were most prominent, alongside ascr#18 and ascr#22, ascarosides with 11- and 13-carbon side chains, respectively (Fig 4, S2 Table).

The very large number of metabolites affected by loss of *acox-1* prevented a comprehensive examination of each of the metabolites. Nevertheless, we used a combination of chemical, genetic, and metabolite add-back experiments to broadly investigate the noted metabolomics changes. We began by considering the possibility that perturbations in ascaroside biosynthesis underlie the feeding defect observed in *acox-1* mutants. Originally identified as constituents of the dauer pheromone, ascarosides are a large class of excreted small molecules that regulate development and behavior and whose synthesis can be influenced by metabolic status [51,52]. The acyl-CoA thiolase, DAF-22, catalyzes the terminal step in peroxisomal β-oxidation and plays an essential role in shortening the fatty acid–like side chains of ascarosides [53]. Despite lacking ascarosides, we found that *daf-22* mutants exhibit wild-type feeding rates and are still responsive to the feeding increasing effects of serotonin, suggesting that ascaroside biosynthesis and serotonergic feeding regulation are independent of one another (S3A Fig). Although a number of ascaroside species were strongly reduced in *acox-1* mutants, we detected a nearly 30-fold accumulation of ascaroside #18 (ascr#18). To determine if an accumulation of ascr#18 underlies the suppressed feeding response of *acox-1* mutants, we treated wild-type animals with ascr#18 and serotonin. Rather than suppressing feeding, ascr#18 dramatically increased pharyngeal pumping rates of wild-type animals in a manner that was additive with serotonin (S3B Fig). To assess the contribution of the highly elevated levels of ascr#18 or other ascarosides to the basal feeding rates of *acox-1* mutants, we treated *acox-1* mutants with *daf-22* RNAi. This manipulation had no effect pharyngeal pumping of *acox-1* mutants (S3C Fig). In addition, we find that *acox-1* mutants also increase their feeding rates upon treatment with exogenous ascr#18 (S3D Fig). Thus, despite the significant increase in the abundance of ascr#18 upon loss of *acox-1*, this ascaroside does not play an obvious role in basal feeding rates of *acox-1* mutants or the failure of these mutants to respond to serotonin.

Thiamine and thiamine derivatives were also significantly accumulated in *acox-1* mutants. We next asked if thiamine supplementation was sufficient to block the feeding-enhancing effects of serotonin, though did not find this to be the case (S3E Fig). As in mammals, *C*. *elegans* does not produce thiamine and obtains this essential cofactor from its diet [54]. Given the pervasive changes to *C*. *elegans* metabolism upon loss of *acox-1*, we speculate that thiamine accumulation in these mutants reflects a general down-regulation of enzyme activities that use thiamine derivatives as cofactors.

We next turned our attention to the finding that loss of *acox-1* perturbs the biosynthesis of ethanolamine-containing lipid species. Most prominently, we find that DAGPE synthesis is strongly reduced in *acox-1* mutants. DAGPEs likely represent intermediates in the biosynthesis of *N*-acylethanolamines (NAEs), a diverse family of signaling molecules with a range of biological roles, including in nutrient sensing and mammalian appetite regulation [55–57]. This family of lipids includes the mammalian orexigenic endocannabinoid arachidonoyl ethanolamide (AEA) and anorectic factor oleoyl ethanolamide (OEA) [58]. To test if a reduction in NAEs contributes to ACOX-1–mediated feeding regulation, we inactivated fatty acid amide hydrolase (FAAH-1), an enzyme involved in the hydrolytic degradation of NAEs. We found that inhibition of FAAH-1, which was shown to increase endogenous NAE abundance [59], stimulates pharyngeal pumping rates of both wild-type and *acox-1* mutants (S3F Fig). Thus, in *C*. *elegans* as in mammals, NAE signaling regulates feeding behavior. These data suggest that reductions in NAEs may contribute to the failure of *acox-1* mutants to increase their feeding rates in response to serotonin. However, this assertion could not be definitively established, as the feeding increasing effects induced by elevated NAEs were not restricted to *acox-1* mutants.

### ACOX-1–mediated regulation of serotonergic feeding circuits requires EGL-2 activity

**Fig 5:**
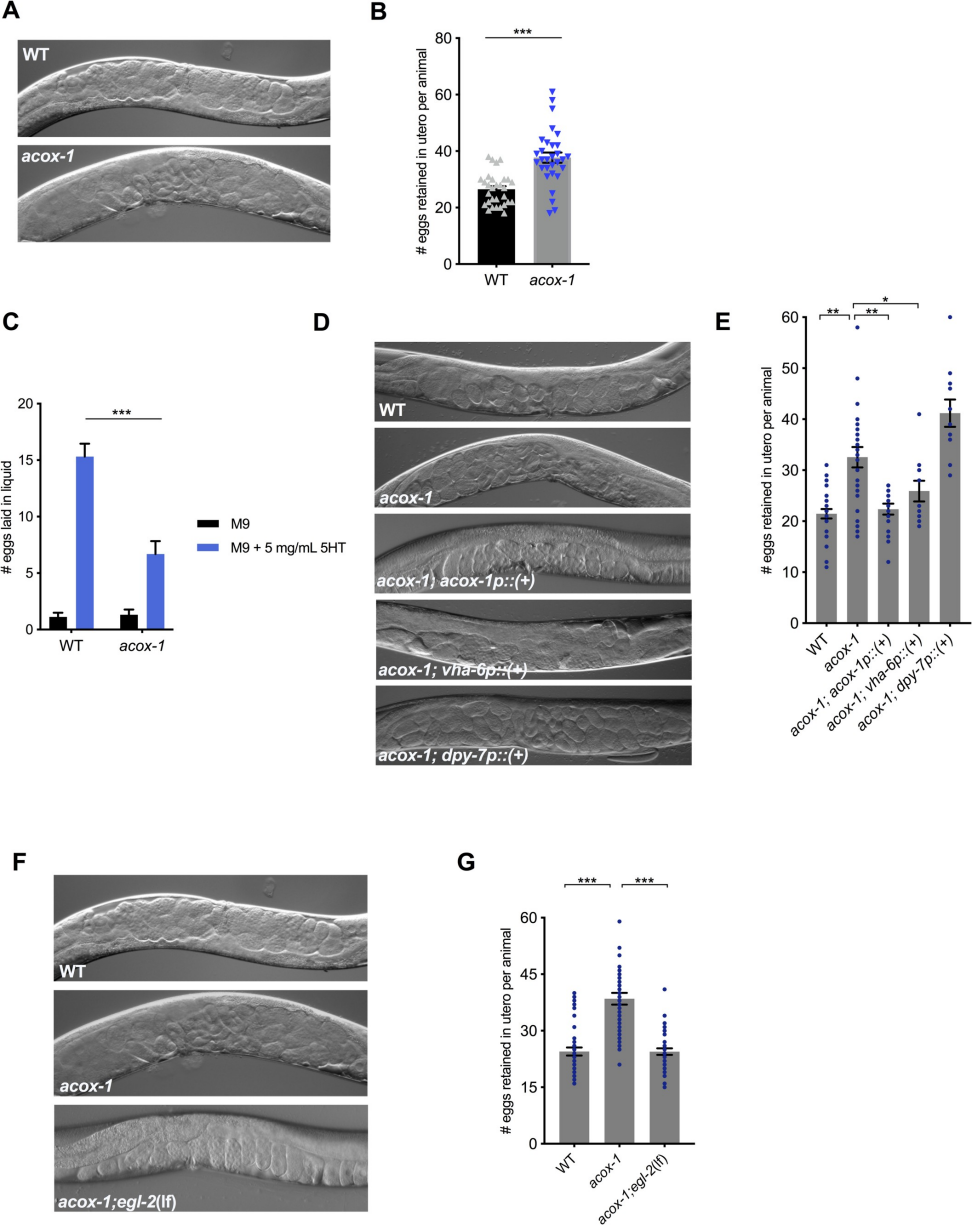
*acox-1* mutants exhibit egg-laying defects. **(A-B*)*** *acox-1(ok2257)* mutants accumulate significantly more eggs in utero than wild-type animals. Representative DIC images **(A)** and quantification **(B)** of eggs retained in utero. Error bars indicate ±SEM from mean, *n* = 30 animals per genotype. ***p < 0.001 unpaired Student *t* test. **(C)** *acox-1* animals are less responsive to the egg-laying inducing effects of serotonin. Egg-laying response of wild-type and *acox-1* in control buffer (M9) or 5 mg/mL serotonin in M9 buffer. Data represent the number of eggs released per animal after a 20-minute exposure to vehicle or drug. Error bars represent ±SEM from mean *n* = 10 animals per condition, ***p < 0.001 ANOVA (Sidak). **(D-E)** Reconstitution of *acox-1* gDNA under an intestine-specific promoter (*vha-6p*) but not an epidermal-specific promoter (*dpy-7p*) restores egg-laying capacity to *acox-1* mutants. Representative DIC images **(D)** and quantification **(E)** of eggs retained in utero. Error bars indicate ±SEM from mean, *n* > 10 animals per genotype, *p < 0.05, **p < 0.01 unpaired Student *t* test, against a wild type. **(F-G)** Loss of *egl-2* rescues *acox-1* egg-laying defects. Representative DIC images of each genotype **(F)** and quantification **(G)** of eggs retained in utero. Error bars indicate ±SEM from mean, *n* = 45 animals per genotype. ***p < 0.001 unpaired Student *t* test. See S1 Data for underlying data. *acox-1*, acyl-coenzyme A oxidase 1; DIC, differential interference contrast; gDNA, genomic DNA; WT, wild-type; 5HT, 5-hydroxytryptamine.

We next sought to identify the neural components that link changes elicited by loss of *acox-1* to serotonergic mechanisms of feeding. The clue that guided us towards an answer emerged unexpectedly by following another phenotype that we had noted in *acox-1* mutants. Relative to wild-type animals, *acox-*1 mutants hold more eggs in utero and lay embryos at a later developmental stage despite having similar brood sizes (Fig 5A and 5B, S4A and S4B Fig). This was intriguing because serotonin is also a key modulator of egg-laying behavior [60]. Serotonin controls the excitability of the egg-laying neuromuscular circuit and governs the activity and timing of egg laying in response to various sensory cues [10,61,62]. We found that *acox-1* mutants were markedly less responsive to exogenous serotonin and resistant to the effects of fluoxetine, a serotonin reuptake inhibitor consistent with a reduced response to the excitatory effects of serotonin at the level of the neuromuscular junction (Fig 5C, S4C Fig). As in the context of feeding, reconstitution of *acox-1* in the intestine but not the epidermis also rescued egg-laying defects of these mutants (Fig 5D and 5E).

The egg-laying neuromuscular circuit has been extensively studied in *C*. *elegans* and it is well established that egg-laying behavior is strongly influenced by potassium channel activity [63]. Potassium channels are a highly diverse and evolutionarily conserved family of proteins that modulate cellular excitability by regulating the flow of potassium ions (K^+^) across cellular membranes [64,65]. Gain-of-function mutations in distinct potassium channels have been shown to reduce the excitability of neurons or vulval muscles, in turn causing egg-laying defects [66–68]. We therefore considered the possibility that aberrant potassium channel activity contributes to the egg-laying dysfunction in *acox-1* mutants. We conducted an RNAi screen of potassium channels with documented roles in egg-laying and assessed their capacity to rescue the egg-laying defect in *acox-1* mutants. RNAi knockdown of the EGL-2 potassium channel normalized egg-laying responses in *acox-1* mutants without influencing baseline egg-laying rates (S4D and S4E Fig). To validate our RNAi results, we crossed *acox-1* mutants with *egl-2(lf)* mutants and found that double mutants resembled wild-type animals in their egg-laying responses (Fig 5F and 5G, S4B Fig). *egl-2* encodes an *ether-a-go-go* (EAG) voltage-gated potassium channel that has been shown to modulate the excitability of neuromuscular circuits in response to starvation states, suggesting they may more generally serve as a link between internal nutrient status and neuronal activity [69–71].

**Fig 6:**
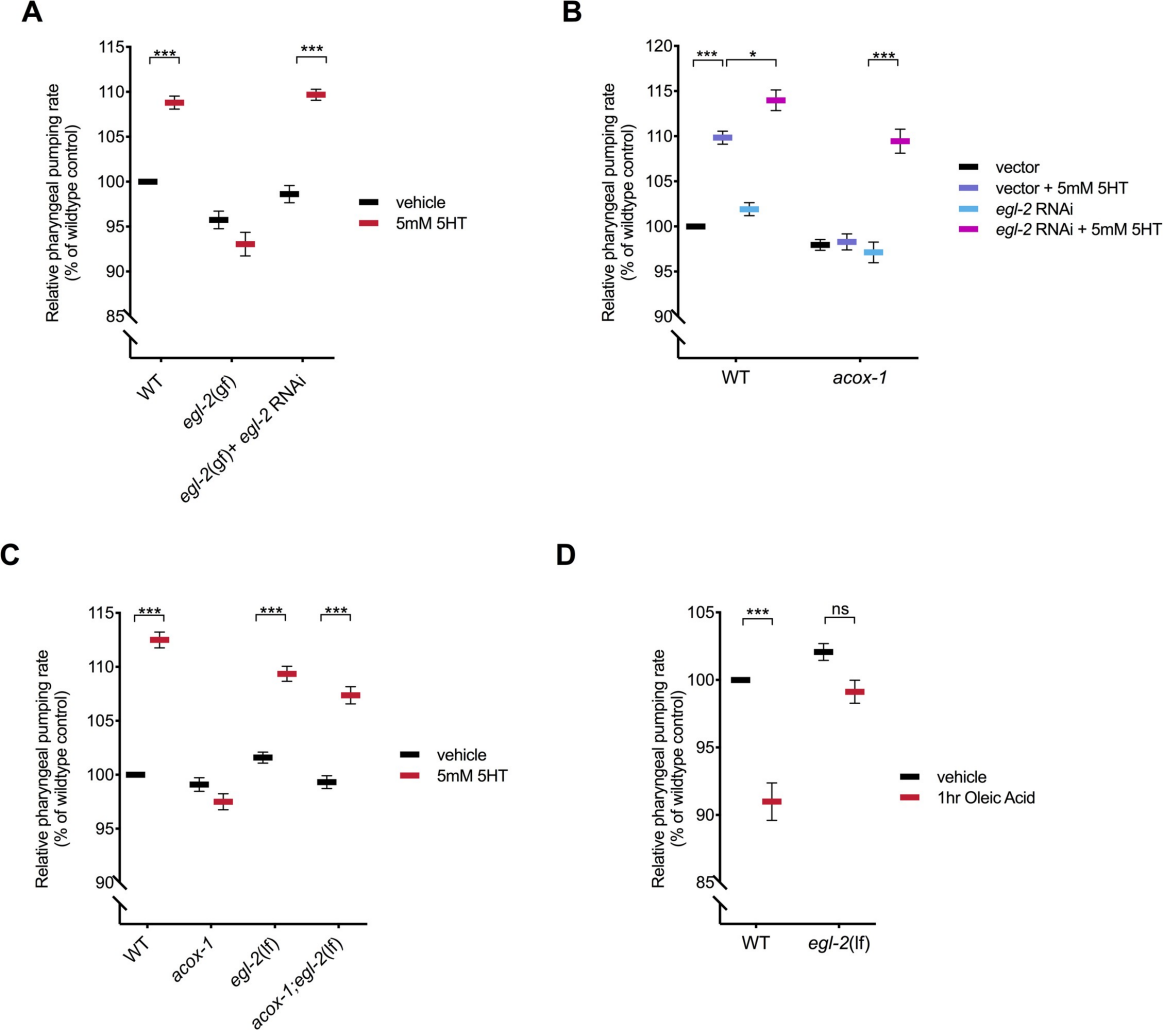
ACOX-1–mediated regulation of serotonergic feeding responses requires the EGL-2 K^+^ channels. **(A)** Relative pharyngeal pumping rates of wild-type, *egl-2(n698)*, and *egl-2* RNAi-treated *egl-2(n698)* mutants on vehicle or 5 mM 5HT–containing plates. **(B)** Relative pharyngeal pumping rates of wild-type and *acox-1(ok2257)* mutants treated with vector RNAi or *egl-2* RNAi and vehicle or 5 mM 5HT. **(C)** Pharyngeal pumping rates of wild-type, *acox-1(ok2257)*, *egl-2(rg4)*, *acox-1(ok2257);egl-2(rg4)* mutants treated with vehicle or 5 mM 5HT. Error bars indicate ±SEM from normalized mean, *n* = 20 animals per condition. *p < 0.05, ***p < 0.001, ANOVA (Tukey). **(D)** EGL-2 is required for animals to reduce feeding in response to oleic acid. Animals were exposed to 1 mM oleic acid for one hour before feeding was assayed. *n* > 15 per strain, error bars indicate ±SEM from normalized mean, ***p < 0.001, ANOVA (Tukey). All feeding data are normalized to vehicle-treated wild-type animals and are presented as a percentage of wild-type rates. See S1 Table and S1 Data for underlying data. ACOX-1, acyl-coenzyme A oxidase 1; EGL-2, EGg-Laying defective 2; ns, not significant; RNAi, RNA interference; WT, wild-type; 5HT, 5-hydroxytryptamine.

To determine if EGL-2 plays a role in ACOX-1–mediated feeding regulation, we took advantage of gain-of-function mutations in *egl-2*. These mutations cause a negative shift in the voltage dependence of these channels, leading to reduced excitatory capacity of cells in which they are expressed [72,73]. It has been previously reported that *egl-2(gf)* mutants are resistant to the egg-laying inducing effects of serotonin [74]. We find that *egl-2(gf)* mutants are also resistant to the feeding-enhancing effects of serotonin and thus mimic *acox-1* mutants in their blunted responses to the excitatory effects of serotonin signaling in the context of both egg laying and feeding (Fig 6A). Both the egg-laying and pharyngeal pumping phenotypes of *egl-2(gf)* mutants can be fully suppressed by *egl-2* RNAi knockdown (Fig 6A, S5A Fig). Similarly, RNAi inactivation of *egl-2* restores the ability of *acox-1* mutants to elevate their pumping rates upon exposure to serotonin signaling but without affecting the basal pumping rate in *acox-1* mutants (Fig 6B). Validating the RNAi results, we find that *acox-1;egl-2(lf)* double mutants exhibit wild-type feeding responses to exogenous serotonin (Fig 6C). Finally, we find that the feeding-reducing effects of oleic acid also require *egl-2* (Fig 6D). Together, these results suggest that an EGL-2 containing circuit can serve as a regulatory link between metabolic status and feeding behavior.

### Body cavity neurons link ACOX-1 activity to serotonergic regulation of egg laying and feeding

**Fig 7:**
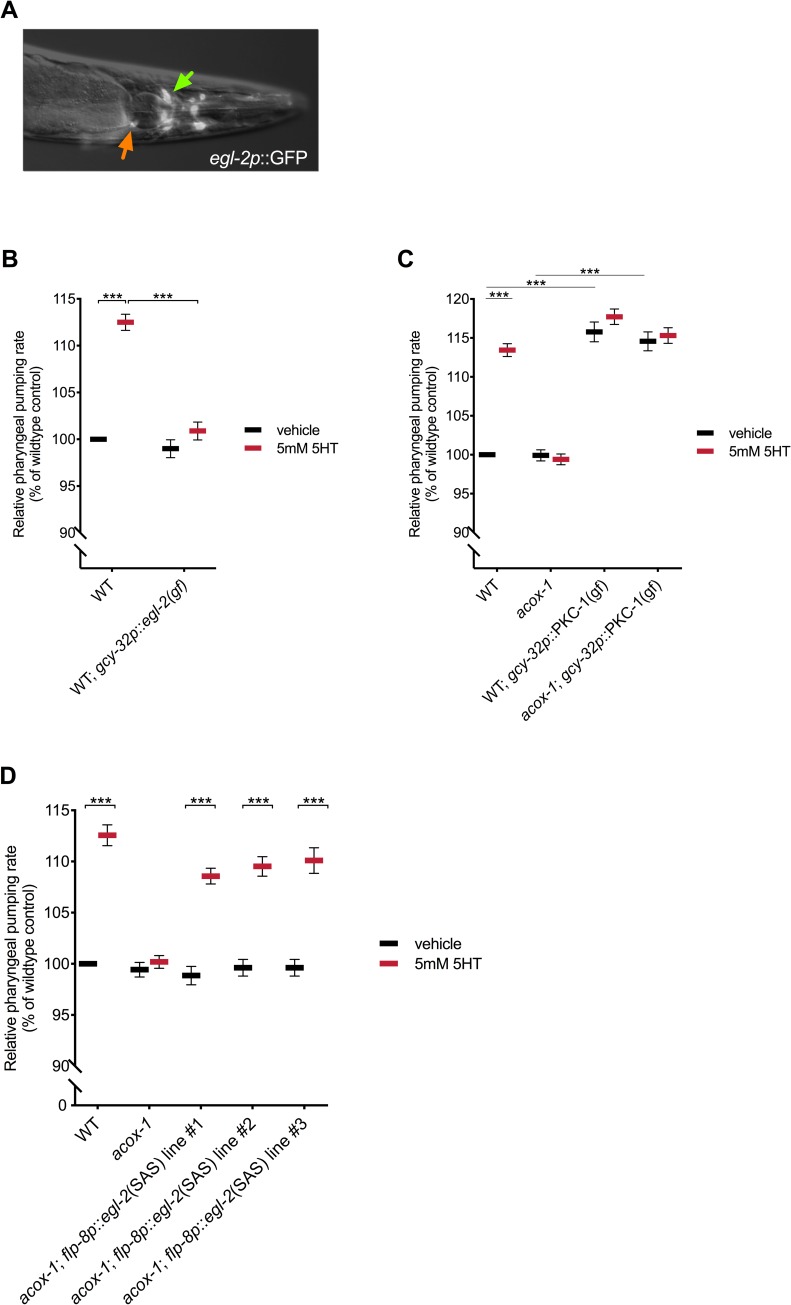
Body cavity neurons modulate feeding behavior. **(A)** EGL-2 is expressed in a limited subset of sensory neurons. Epifluorescent image of a transgenic animal expressing *egl-2p*::*gfp* transcriptional reporter. Orange and green arrows indicate the AQR and URX body cavity neurons, respectively. **(B)** Animals expressing *egl-2(gf)* in body cavity neurons do not elevate feeding in response to 5mM 5HT. Relative pharyngeal pumping rates of wild-type and *gcy-32p*::*egl-2(gf)* expressing animals treated with vehicle or 5 mM 5HT. **(C)** Constitutive activation of synaptic release from body cavity neurons stimulates feeding. Relative pharyngeal pumping rates of wild-type, *acox-1(ok2257)*, CX10386 *gcy-32p*::*pkc-1(gf)*, and *acox-1;* CX10386 *gcy-32p*::*pkc-1(gf)* animals treated with vehicle or 5 mM 5HT. **(D)** Cell-specific RNAi of *egl-2* in URX neurons is sufficient to rescue feeding defects of *acox-1* mutants. Sense and antisense (SAS) transgenes targeting *egl-2* were expressed under the control of *flp-8*, a URX specific promoter. Relative pharyngeal pumping rates of wild-type, *acox-1(ok2257)* and three independent lines of *acox-1*; *flp-8p*::*egl-2*(SAS) animals treated with vehicle or 5 mM 5HT. In **(B)** and **(C)**, feeding data are normalized to vehicle-treated wild-type animals and are presented as a percentage of wild-type animals. Error bars indicate ±SEM from normalized mean, *n* = 15 animals per condition. ***p < 0.001 two-way ANOVA (Tukey). All feeding data are normalized to vehicle-treated wild-type animals and are presented as a percentage of wild-type rates. See S1 Table and S1 Data for underlying data. *acox-1*, acyl-CoA oxidase 1; EGL-2, EGg-Laying defective 2; *flp-8*, FMRF-Like Peptide 8; SAS, sense and antisense; WT, wild-type; 5HT, 5-hydroxytryptamine.

In hermaphrodites, *egl-2* is expressed in a limited subset of sensory neurons, including AFD, ALN, AQR, ASE, AWC, BAG, IL2, PLN, PQR, and URX [72]. We were intrigued by the expression of *egl-2* channels in body cavity neurons (AQR, PQR, and URX), as these are the only neurons with dendritic projections within the pseudocoelom, the rudimentary circulatory fluid utilized by *C*. *elegans* (Fig 7A) [75]. Given their unique anatomic position, these neurons are hypothesized to mediate bidirectional communication between the nervous system and peripheral tissues, as they have the capacity to both release and detect circulating signals. Interestingly, neural serotonin signaling is thought to promote intestinal fat metabolism through the release of a neuroendocrine signal from URX neurons [23,24]. We find that targeted expression of *egl-2(gf)* in only the body cavity neurons is sufficient to block the feeding-increasing effects of serotonin, mimicking the effects of loss of peripherally expressed *acox-1* (Fig 7B).

To our knowledge, body cavity neurons have not previously been implicated in the regulation of feeding behavior. To further study their role, we examined the effect of prolonged activation of body cavity neurons on pharyngeal pumping. Activation of protein kinase C (PKC-1) has been shown to promote synaptic transmission and neuropeptide release from expressing neurons [76]. We examined the feeding responses of wild-type and *acox-1* mutants expressing constitutively active protein kinase C [PKC-1(gf)] in body cavity neurons and found that this manipulation strongly stimulates pharyngeal pumping in both wild-type and *acox-1* mutants. Importantly, this feeding enhancement could not be further elevated by addition of serotonin, suggesting a common feeding regulatory circuit (Fig 7C). As genetic inhibition of body cavity neurons by *egl-2(gf)* did not modulate baseline feeding rates, our data suggest that these neurons likely fine-tune feeding responses rather than governing basal feeding behavior.

The above genetic experiments suggested that inhibition of body cavity neurons, particularly that of URX, mimics the effect of loss of *acox-1*, while activation of these neurons restores serotonin responsiveness to *acox-1* mutants. To more directly assess the effects of ACOX-1 activity on body cavity neuron function, we measured intracellular Ca^2+^ transients from the URX body cavity neurons using the genetically encoded calcium reporter GCaMP5K [77,78]. One issue with this approach is that under standard laboratory conditions, there is little detectable GCaMP5K activity in these neurons; however, URX neurons play a well-documented role in oxygen sensing and a robust and rapid activation of the GCaMP5K reporter is seen upon a change in oxygen levels from 10% to 21% oxygen (S6A and S6C Fig) [79]. We observed no significant difference in URX responses in wild-type and *acox-1* mutants at 10% oxygen, a concentration at which the tonic URX neurons are held in the “off” state (S6B and S6F Fig) [80]. However, whereas URX neurons in wild-type animals robustly activate at 21% oxygen as previously documented, O_2_-evoked calcium transients were dramatically inhibited in *acox-1* mutants (S6B and S6D Fig). This reduction in maximal activation (FΔ/F_o_) could not be attributed to altered promoter activity or drift in expression across the tested lines as the level of a co-expressed *flp-8p*::mCherry reporter was not measurably different from *acox-1* mutants (S6E and S6G Fig). Moreover, loss of *acox-1* did not have a broad dampening effect on neural activity as another interneuron, RIM, was as responsive to another stimulus, pairing of butanone with food, as wild-type animals (S6H and S6I Fig). These initial findings were consistent with the notion that loss of *acox-1* decreases the sensitivity of URX neurons to excitatory stimuli. However, neither loss of *egl-2* nor intestine-specific reconstitution of *acox-1*, two manipulations that rescued the feeding and egg-laying phenotypes of *acox-1* mutants, restored O_2_-evoked calcium transients to *acox-1* mutants (S7 and S8 Figs). One interpretation of these results is that O_2_-evoked calcium transients do not provide an appropriate proxy for neural activities that are relevant to feeding behavior and egg laying.

To further investigate the role of URX body cavity neurons in the context of *acox-1* mutants, we returned to genetic strategies. We took advantage of a previously described cell-specific RNAi approach to specifically knock down the expression of EGL-2 channels in URX neurons [81]. Using a *flp-8* promoter, *egl-2* RNAi was targeted to URX neurons in *acox-1* mutants. This manipulation restored the ability of *acox-1* mutants to increase their feeding rates in response to serotonin and their normalized egg-laying capacities (Fig 7D). Together, these studies provide strong genetic support for the notion that the URX neurons serve to link metabolic changes caused by loss of peripheral *acox-1* to serotonergic responses.

## Discussion

Animals adopt distinct behavioral and physiological states in response to changes in internal metabolic status. In this study, we show that peripherally generated signals act to modulate neurally regulated processes in *C*. *elegans*. Specifically, we found that loss of an intestinal peroxisomal acyl-CoA oxidase leads to the production of interoceptive signals of metabolic status that modulate serotonergic regulation of feeding and egg laying. These signals are generated when acyl-CoAs that would normally be destined for peroxisomal β-oxidation via ACOX-1 are redirected into other pathways, resulting in a vast change to the animal’s metabolome despite a very modest transcriptional effect. The combination of spatially restricted studies of *egl-2* loss- and gain-of-function as well as modulation of neural activity by PKC activation are consistent with a model in which loss of peripherally expressed *acox-1* results in generation of metabolic signals that ultimately blunt the activity of body cavity neurons, particularly that of URX. While our metabolomics analyses identified several metabolic signals that affect feeding behavior and whose levels are altered upon loss of *acox-1*, the precise identities of the peripherally generated signals that modulate the activity of body cavity neurons are not yet known. Similarly, the mechanisms by which body cavity neurons then intersect with serotonergic circuits of feeding and egg laying remain to be determined.

Our genetic and pharmacological analyses suggest that the feeding regulatory response begins with an accumulation of fatty acyl-CoA species. Physiological conditions in which generation of acyl-CoAs exceeds their utilization are predicted to occur during periods of nutrient excess, and thus the feeding-reducing effects of these signals are consistent with satiety-like signals. In mammals, pharmacologic or dietary manipulations that lead to elevated circulating fatty acyl-CoA levels also inhibit food intake [82,83]. Fatty acyl-CoAs are short-lived species that are substrates for numerous enzymatic pathways, and it is unclear in mammals whether the anorectic effect is mediated directly by specific fatty acyl-CoAs or downstream metabolic derivatives [84]. While we could not measure an obvious increase in acyl-CoA pools extracted from *acox-1* mutants, our metabolomic analyses revealed many hundreds of differentially expressed features in *acox-1* mutants, the majority of which remain structurally unidentified. The enormous metabolomic change made it unrealistic for us to pinpoint the precise metabolic species that explain the effects of loss of *acox-1* on serotonergic signaling. Nevertheless, our metabolomics analyses revealed several classes of compounds, including a variety of phospholipid species, that may underlie the noted behavioral changes. Several NAEs have well-known effects on mammalian feeding behavior and mood [57,85,86]. Their identification here highlights the deep evolutionary origins of the links between metabolic state and neural mechanism that influence behavior.

We found that body cavity neurons, most prominently URX, serve as key components of the sensory circuit linking peripheral metabolic information with feeding behavior. Body cavity neurons are known to regulate oxygen sensing and social aggregation behaviors, although to our knowledge they have not been implicated in regulation of pharyngeal pumping [87–89]. Given their unique anatomical placement, these neurons have long been hypothesized to facilitate bidirectional communication between the nervous system and peripheral tissues, particularly in the context of energy homeostasis. Several lines of evidence, in addition to our findings, offer experimental credence to this hypothesis. First, these neurons are in direct synaptic contact with environment sensing neurons like ADF and release modulatory signals to influence peripheral processes such as body growth, life span control, and lipid metabolism [77,90,91]. Second, these neurons have the capacity to sense internal metabolic cues and integrate this information with external cues of nutrient availability to orchestrate cohesive and context-appropriate physiological responses [77]. Lastly, our genetic data and a recent study suggest that internal nutrient sensing pathways modulate the activity of these neurons to regulate nutrient-dependent behavioral states [92]. Although we were initially intrigued to find that loss of *acox-1* dampens the oxygen-evoked GCaMP responsiveness of URX neurons, it is currently unclear whether this readout is an appropriate proxy for the neural activities that are relevant to feeding rate and egg laying. This is because manipulations that restored wild-type feeding and egg laying to *acox-1* mutants failed to similarly restore the full extent of oxygen-evoked GCaMP responsiveness to these neurons. Although changes to oxygen-evoked URX calcium transients have been reported by multiple groups, we are unaware of any examples from the literature where such a change could be rescued by cell nonautonomous manipulations as we attempted in this study.

An important future challenge is to precisely determine how the body cavity neurons, including URX, sense the vast metabolic changes caused by loss of peripheral *acox-1*. In principle, it is possible that some of the peripheral metabolites leave their intestinal sites of generation to directly act on the URX neurons. Alternatively, it is possible that an endocrine response originating in the intestinal cells communicates the metabolic status of the intestine to the URX neurons. Regardless of which model may ultimately be valid, our findings point to *egl-2* as a modulator of URX activity. EGL-2 is a *C*. *elegans* potassium channel with close homology to human EAG K^+^ channels [72,93]. Mammalian EAG channels have not yet been implicated in the control of nutrient-dependent behavioral plasticity; however, many potassium channels play well-documented roles in metabolic sensing and energy homeostasis. For example, hypothalamic Kir6.2 K_ATP_ channels are responsive to circulating glucose levels and regulate appetite and glucose homeostasis [94,95]. Ketogenic diets, which increase circulating ketone body levels, have been shown to suppress the excitability of Gamma-Aminobutyric Acid (GABA)ergic neurons and reduce seizure susceptibility in a K_ATP_ channel–dependent manner [96,97]. Numerous metabolites, including phospholipids, polyunsaturated fatty acids, eicosanoids, fatty acyl-CoAs, and oxygen, can directly bind certain potassium channels to modulate channel activity [98], although only a few metabolic modifiers of EAG channels have been identified. Arachidonic acid and other polyunsaturated fatty acids have been shown to enhance the activity of mammalian EAG channels, while phosphatidylinositol 4,5-bisphosphate (PIP2), a phospholipid component of plasma membranes, has been shown to inhibit these channels [99]. Additionally, select plant-derived flavonoids have also been shown to modulate EAG channel dynamics [100,101]. The physiological relevance of these metabolite-channel interactions is poorly understood. Alternatively, metabolic information can be coupled to potassium channels, including EAG channels, by indirect signaling events involving second messengers like Ca^2+^ and cAMP, neurotransmitters, or posttranscriptional modifications [102,103].

In both mammals and *C*. *elegans*, elevated serotonin signaling is associated with fat loss [12,104]. In mammals, this has been primarily attributed to the anorectic effects of serotonin. By contrast, detailed analysis of serotonergic effects in *C*. *elegans* revealed that distinct molecular mechanisms underlie the fat and feeding effects of serotonin and that serotonin-induced fat reduction in *C*. *elegans* is primarily driven by up-regulation of peripheral mechanisms of triglyceride lipolysis and β-oxidation [12,23,24]. Although they are generally overshadowed by the feeding behavioral data, the existing data indicate that serotonin also causes an increase in metabolic rate and increased fat oxidation in mammals [22,105]. In this study, we found that if β-oxidation pathways cannot utilize the influx of acyl-CoAs that are generated upon mobilizing stored triglycerides, a homeostatic signal is generated to blunt serotonergic effects, including effects on feeding. The complexity of the mammalian fat and feeding pathways has made it difficult to pinpoint the precise mechanisms through which serotonin levels act as satiety signals. Our findings raise the possibility that in both worms and mammals, increasing serotonin signaling may result in peripheral metabolic changes that, in turn, feed back to the nervous system to modulate food intake.

## Materials and methods

### Worm strains and general maintenance

*C*. *elegans* strains were cultured under standard growth conditions [106]. The Bristol N2 strain was used as wild type in all experiments, and the following mutant alleles and transgenic strains were analyzed: *acox-1(ok2257)*, *aak-2(ok524)*, *egl-2(rg4)*, *egl-2(n2656)*, *daf-22(m130)*, CX10386 *kyEx2491*[*gcy-36p*::*pkc-1(gf)*::SL2::GFP; *ofm-1p*::*dsRed*], SSR1070 *flp-8p*::*mCherry; flp-8p*::*GCaMP5K*. When generating double mutants, genotypes were confirmed by PCR or sequencing. Unless stated otherwise, animals were cultured on NGM agar plates with OP50 *E*. *coli* at 20°C. For all experiments, worms were plated as synchronized L1 populations after hypochlorite treatment of gravid adults.

### Plasmid construction and transgenesis

Plasmids were constructed using Gateway Cloning Technology (Life Technologies, Carlsbad, CA). Promoter regions were amplified from wild-type gDNA using Phusion DNA polymerase (New England Biolabs, Ipswich, MA) and subcloned into the pDONR-P4-P1R Gateway entry vector by BP recombination. Oligonucleotides used in this study are listed in S3 Table. For *acox-1* tissue-specific rescue lines, full *F08A8*.*1* genomic coding sequence was amplified from wild-type gDNA and was subsequently subcloned into the P221 Gateway entry vector by BP recombination. Transgenic animals were generated by injecting purified plasmids into the gonads of wild-type or mutant animals. Unless otherwise specified, transgenic rescue constructs and an *unc-122p*::gfp co-injection marker were injected at a concentration of 50 ng/μL. At least two independent and stably expressing lines were maintained and analyzed. To reduce the expression of *egl-2* specifically in URX neurons, we used a cell-specific RNAi approach using the *flp-8* promoter as described before [77,81]. Sense and antisense sequences targeting the *egl-2* gene were amplified from the RNAi clone that was used for the RNAi feeding experiment. The vectors for the sense and antisense expression of *egl-2* under the *flp-8* promoter were generated using NEBuilder HiFi DNA Assembly cloning kit (New England Biolabs, Ipswich, MA) and injected into *acox-1* mutants at 30 ng/μL each, together with 50 ng/μL of the selection marker *unc-122p*::gfp. Three independent lines were assayed.

### Serotonin, fluoxetine, oleic acid, and triacsin C treatments

Stock solutions of serotonin hydrochloride (TCI America, Portland, OR, S0370) and fluoxetine hydrochloride (Matrix Scientific, Columbia, SC, 047891) were prepared in water. For feeding experiments, synchronized L1 animals were grown on OP50 plates containing 5 mM serotonin and assayed at the day 1 adult stage. To determine egg-laying responses, animals were exposed to 0.5 mg/mL fluoxetine in M9 buffer or 5 mg/mL serotonin in M9 buffer for 20 minutes prior to counting released eggs. Oleic acid and triacsin C treatments were conducted as previously described [12]. Briefly, oleic acid (Sigma-Aldrich, St. Louis, MO, O1383) was solubilized in 45% (w/v in dH_2_O) 2-hydroxypropyl-ß-cyclodextrin (Sigma-Aldrich, St. Louis, MO, H5784) to 1 M and then added to OP50 plates to a final concentration of 1 μM. Triacsin C (Enzo Life Sciences, Farmingdale, NY, BML-EI218) was used at a final concentration of 1 μM (0.0002% DMSO) on OP50 plates.

### Pharyngeal pumping

Contractions of the posterior pharyngeal bulb were counted during a 10-second interval using a Zeiss M2-Bio microscope. In each experiment, vehicle- or vector-treated wild-type pumping rates were normalized to 100 and data are presented as the percentage of wild-type rates. The unnormalized data are listed in S1 Table. Unless otherwise stated, 15 day one animals were assayed per condition for each genotype, and each experiment was repeated at least 3 times. For measurements on fasted then refed animals, ad libitum fed day 1 adults were washed 3 times in S-basal buffer to remove residual *E*. *coli* and subsequently placed on sterile NGM plates. Animals were fasted on plate for the indicated time, then transferred to either vehicle (OP50 *E*. *coli*) or treatment (5 mM serotonin + OP50) containing plates for 90 minutes to assay post-fast feeding responses. In conditions in which DMSO was used as a solvent, the DMSO concentration did not have an effect on pumping rate.

### RNAi treatment

Overnight cultures of HT115 *E*. *coli*–containing RNAi plasmids were induced with 6 mM IPTG for 4 hours at 37°C. Cultures were concentrated 2× and added to RNAi plates containing IPTG, carbenicillin, and tetracyclin. Synchronized L1 animals were added to plates and grown for 3 days at 20°C, and assayed as day 1 adults.

### Microscopy

Animals were mounted on 2% agarose pads, paralyzed with NaN_3_ and imaged using a Zeiss Axioplan 2 microscope with a 16× (0.55 NA) oil immersion objective. DIC images of eggs retained in utero were acquired from animals at the Day 1 adult stage.

### Acyl-CoA quantification

We adapted an HPLC-based acyl-CoA extraction, derivatization, and quantification protocol from Larson and colleagues, 2008 [43]. Briefly, 15,000 synchronized L1s were grown in liquid S-medium culture at 20°C on a rotary shaker. Animals were grown to the L4 stage and washed 3× in S-Basal, then snap-frozen in liquid nitrogen and stored at −80°C until further processing. To prepare lysates, pellets were thawed on ice and resuspended in 300 μL of freshly prepared extraction buffer (2 mL 2-propanol, 2 mL pH 7.2 50 mM KH_2_PO_4_, 50 μL glacial acetic acid, 80 μL 50 mg/mL BSA). Approximately 500 mg of 0.5-mm-diameter zirconium oxide beads (NextAdvance, Troy, NY) were added to each sample, and animals were lysed using a bead beater (5 cycles: 30 seconds ON, 1 minute OFF) at 4°C. Lysates were separated from beads by pipet and an aliquot was preserved for protein quantification (Bio-Rad, Hercules, CA). Lysates were then washed of lipids and pigments in 200 μL petroleum ether (40–60°C) saturated 1:1 with 2 propanol:water. A total of 5 μL of saturated (NH_4_)_2_SO_4_ was added to samples before extracting acyl-CoAs with 600 μL 2:1 methanol:chloroform. Samples were vortexed and incubated at room temperature for 30 minutes before centrifugation. Supernatants were transferred to glass tubes and dried at 40°C in a GeneVac (approximately 2 hours). Once dry, samples were reconstituted in 55 μL of chloroacetaldehyde derivatizing reagent (0.15 M sodium citrate, 0.5% SDS [w/v], 0.5 M chloroacetaldehyde, pH 4) and incubated in an 80°C water bath for 20 minutes. Samples were again clarified by centrifugation before being transferred to HPLC sample tubes. A total of 20 μL of each sample was injected into an equilibrated 4.6 mm × 150 mm, 5 μm silica particle, 100 Å pore size C18 reversed-phase HPLC column (Phenomenex Luna, Torrance, CA). A linear gradient of 0.25% (v/v) triethylamine in water and 90% (v/v) acetonitrile in water at a 0.5 mL/minute flow rate was used to elute acyl-CoAs. The full elution protocol is described in [43]. Derivatized acyl-CoAs were detected using a fluorimeter with a flash rate of 100 Hz and with excitation wavelength at 230 nM, emission wavelength at 420 nM, and slid width at 20 nM. Peak areas were integrated and quantified using Agilent ChemStation software.

### RNA-seq sample preparation

Total RNA was extracted from approximately 10,000 synchronized L4 animals using the standard Trizol, chloroform, isopropanol protocol with on-column DNAse digest. Sample quality and quantity were assessed on the Agilent Bioanalyzer using RNA 600 Nano chips, and only samples with an RIN score ≥9 were used for library construction. mRNA was enriched from 1 μg of total RNA using NEXTflex Poly(A) Beads (Bioo Scientific, Austin, TX, NOVA-512979), and strand-specific directional libraries were generated using the NEXTflex Rapid Directional RNA-Seq Kit (Bioo Scientific). For each genotype, samples were prepared from 3 biological replicates and indexed with distinct barcodes. Quality and fragment size distribution of synthesized cDNA libraries were assessed on the Agilent Bioanalyzer using DNA 1000 chips. Library concentrations were quantified using the NEBNext Library Quant Kit (New Englad Biolabs, Ipswich, MA, E7630S), and each library was normalized to 10 nM in TE buffer prior to pooling. Multiplexed libraries were sequenced using 100-bp paired-end reads on the Illumina HiSeq 4000 platform at the UCSF Center for Advanced Technologies.

### RNA-seq analysis

Transcriptomic analyses were performed on the Galaxy Platform [107]. Sequencing reads were filtered and trimmed to remove barcode sequence using fastx_trimmer version 0.0.13 (http://hannonlab.cshl.edu/fastx_toolkit/). Clipped paired-end sequences were aligned to the *C*. *elegans* ce11 reference genome using TopHat version 2.1.1 [108]. Read counts for each gene were quantified using *htseq-count* based on gene annotations from reference annotation WS235 [109]. Differential expression analysis was conducted using the DESeq2 package (3.8) in R(3.4.1) using size factor normalization [110]. *p*-values were adjusted for multiple comparisons using the Benjamini-Hochberg method, and a permissive false discovery threshold of q ≤ 0.1 was applied to identify differentially expressed transcripts.

### Metabolomic sample preparation

Mixed stage worms were grown in liquid and fed OP50 on days 1, 3, and 5 during the 7-day culture period, while shaking at 22°C and 220 rpm. The cultures were centrifuged at 4°C, and worm pellets and supernatant were frozen separately, lyophilized, and each extracted with 35 mL of 95% ethanol at room temperature for 12 hours. The extracts were dried in vacuo, resuspended in 200 μL methanol, and analyzed by LC-HRMS. All cultures were grown in at least 3 biological replicates.

### Mass spectrometric analysis

LC-MS analysis was performed on a Dionex 3000 UHPLC coupled with a ThermoFisher Q Exactive high-resolution mass spectrometer. Metabolites were separated using a water–acetonitrile gradient on Zorbax Eclipse XDB-C18 column (Agilent, Santa Clara, CA, 150 mm × 2.1 mm, particle size 1.8 μm) maintained at 40°C. Solvent A: 0.1% formic acid in water; Solvent B: 0.1% formic acid in acetonitrile. The solvent gradient started at 5% B for 5 minutes after injection and increased linearly to 100% B at 12.5 minutes, and continued at 100% B for 5 minutes. The gradient was rapidly brought down to 5% B (over 30 seconds) and held for 2 minutes for re-equilibration.

The UHPLC-MS data were collected in the profile MS mode, based on instrument specifications. Metabolites were detected as [M-H]^−^ ions or [M+Cl]^-^ adducts in the negative ionization mode (ESI+), or as [M+H]^+^ ions or [M+Na]^+^ adducts in the positive ionization mode (ESI+), using a spray voltage of 3 kV. Compound identities were confirmed based on their high-resolution masses (accuracy <1 ppm), MS/MS fragmentation spectra, and/or comparison with authentic standards. Data analysis was carried out using the Bioconductor package XCMS [47,111]. The matched filter algorithm in XCMS for peak picking in the profile data was used.

### GCaMP imaging of O^2^-evoked transients in URX neurons

Animals were exposed to different oxygen concentrations (10% or 21%) using a microfluidic chamber constructed with the oxygen-permeable poly(dimethylsiloxane) (PDMS) as described [80]. A Valvebank II (AutoMate Scientific, Berkeley, CA) was used to control input from two pressurized premixtures of oxygen and nitrogen containing either 10% oxygen or 21% oxygen. The gas flow rate was measured by a VWRTM traceable pressure meter and set to 0.26 psi. At the time of experiment, an individual day 1 adult animal was picked without using any food and consecutively transferred to two unseeded plates immediately before imaging. The transferred animal was immobilized in S basal containing 5 mM levamisole and transported into the microfluidic chamber via Tygon tubing (Saint-Gobain, Danville, CA). To avoid drying, the animal was constantly submerged in S-Basal buffer while inside the chamber, and GCaMP5K fluorescence was visualized at 40× magnification using a spinning disk confocal microscope (Olympus America, San Jose, CA) with MetaMorph software (version 6.3r7, Molecular Devices). As described earlier, worms were preexposed to 10% oxygen for 5 minutes in the microfluidic chamber [80]. GCaMP5K fluorescence was recorded by stream acquisition for 2 minutes at a rate of 8.34 frames/second with an exposure time of 20 ms using a 12-bit Hamamatsu ORCA-ER digital camera. Each animal was recorded once, and GCaMP5K-expressing neurons were marked by a region of interest (ROI). The change in fluorescent intensity as per neuronal excitation and position of the ROI was tracked using the “Track Objects” function in MetaMorph. To subtract background from the total integrated fluorescence intensity of the ROI, an adjacent ROI was selected in the same image. MATLAB (MathWorks) was used to analyze the data. Fluorescence intensity is presented as the percent change in fluorescence relative to the baseline (ΔF/F_0_). F_0_ was measured in worms exposed to 10% oxygen during the first 9–13 seconds for each recording and calculated as an average over that period. The number of animals used for each condition is denoted in the figures.

### GCaMP imaging of spontaneous transients in RIM neurons

Day 1 wild-type and *acox-1* mutants that express Ex[*cex-1p*::GCaMP3.0; *tdc-1p*::mCherry] were conditioned with 10% butanone for 1 hour and mounted on a 10% agarose pad containing 2 μL of 0.1 mm polystyrene beads followed by immobilization with coverslips. GCaMP imaging for control and conditioned animals was performed as described before [17]. Briefly, fluorescence images were taken for 250 seconds at 10-ms exposure time and 500-ms sampling interval with 2 × 2 binning under magnification of 40×. Acquired pictures were analyzed using image J software (NIH). The integrated fluorescence intensity for each frame was normalized to the baseline values, (F-F_b_)/ F_b_, and summed to obtain a total integrated fluorescence measurement for each sample.

### Statistics

Graphpad Prism 8.0 software package was used to calculate all *p*-values unless otherwise specified. Where only 2 conditions were compared, a two-tailed Student *t* test was performed. ANOVAs with appropriate posttest corrections were used when comparing multiple conditions.

## Supporting information

S1 Fig Related to Fig 1. **(A)** Knocking down *acox-1(F08A8*.*1)* by RNAi suppresses the feeding elevating effects of exogenous serotonin (5 mM 5HT). Animals were treated with RNAi from the L1 stage, and feeding was assayed at day 1 adult stage. Feeding data are normalized to vehicle-treated wild-type animals and are presented as a percentage of wild-type animals. Error bars indicate ±SEM from normalized mean, *n* = 20 animals per strain. ***p < 0.001 ANOVA (Tukey). **(B)** Pharyngeal pumping rates were counted over a longer interval (60 seconds) from video recordings. Error bars indicate ±SEM from normalized mean, *n* = 10 animals per condition. ***p < 0.001 ANOVA (Tukey). See S1 Data for underlying data. **(C)** Loss of *acox-1* does not influence the transcriptional expression of tryptophan hydroxylase (*tph-1*) as measured by qPCR. Error bars indicate ±SEM from mean *n* = 3 independent assays. **(D)** Relative abundance of 5HT in wild-type and *acox-1* mutants, as determined by LC-HRMS. Error bars indicate ±SEM from mean, *n* = 4 independent experiments. (**E**) Loss of *acox-1* does not grossly alter amphid neuron morphology. DiI staining of amphid chemosensory neurons in wild-type and *acox-1* mutants. Images acquired at day 1 adult stage. *acox-1*, acyl-coenzyme A oxidase 1; HRMS, high-resolution mass spectrometry; LC, liquid chromatography; qPCR, quantitative PCR; RNAi, RNA interference; 5HT, 5-hydroxytryptamine.
(TIF)

S2 Fig Related to Fig 3. **(A)** Treating *aak*-2;*acox-1* mutants with 1 μM triacsin C for 60 minutes elevates pharyngeal pumping rates. Day 1 adult animals were treated with to vehicle (0.0002% DMSO) or 1 μM triacsin C for 60 minutes before assessing pharyngeal pumping rates. Error bars indicate ±SEM from normalized mean, *n* = 15 animals per condition. At the concentration used, triacsin C had no effect on feeding rate. **(B)** RNAi-mediated inactivation of distinct ACSs suppress feeding defects in *acox-1(ok2257)* animals. Animals were treated with respective RNAi clones from L1 stage and feeding was assayed at day 1 adult stage. All feeding data are normalized to vehicle and vector RNAi-treated wild-type animals and are presented as a percentage of wild-type rates. Error bars indicate ±SEM from normalized mean, *n* = 15 animals per strain. *p < 0.05, ***p < 0.001 ANOVA (Tukey). See S1 Data for underlying data. CoA, co-enzyme A; RNAi, RNA interference.
(TIF)

S3 Fig Related to Fig 4. **(A)** *daf-22(m130)* animals are still responsive to the feeding elevating effects of 5 mM serotonin, *n* = 10 animals per condition. **(B)** Feeding responses of wild-type animals to ascr#18. Animals were exposed to 1 μM and 5 μM ascr#18 from the L1 stage, and pharyngeal pumping rates were determined at day 1 adult stage. *n* = 15 animals per condition. **(C)** Feeding responses of *acox-1(ok2257)* animals to vector and *daf-22* RNAi, *n* = 15 animals per condition. **(D)** Feeding responses of wild-type and *acox-1* mutants to 5 μM ascr#18, *n* = 15 animals per condition. **(E)** Feeding responses of wild-type animals to thiamine. Animals were exposed to 0.5 μg/μL thiamine and 1.0 μg/μL thiamine from L1 stage, and pharyngeal pumping rates were determined at day 1 adult stage. *n* = 15 animals per condition. **(F)** RNAi-mediated inactivation of FAAH-1 elevates feeding responses of wild-type and *acox-1* mutants. Animals were grown on *faah-1* RNAi from L1 stage and feeding was assayed at day 1 adult stage, *n* = 10 animals per condition. All feeding data are normalized to vehicle-treated wild-type animals and are presented as a percentage of wild-type animals. Error bars indicate ±SEM from normalized mean, ***p < 0.001 two-way ANOVA (Tukey). See S1 Data for underlying data. ascr#18, ascaroside #18; FAAH-1, fatty acid amide hydrolase; RNAi, RNA interference.
(TIF)

S4 Fig Related to Fig 5. **(A)** *acox-1(ok2257)* mutants lay eggs at a later developmental stage than wild-type animals, suggesting that in utero retention time is increased. Histograms indicate the distribution of embryos at each developmental stage. **(B)** There was no significant difference in the total number of progeny between wild-type, *acox-1*, *egl-2*, and *acox-1;egl-2* mutants. **(C)** *acox-1* mutants are less responsive to the egg-laying inducing effects of serotonin. Egg-laying response of wild-type and *acox-1* mutants in control buffer (M9) or 0.5 mg/mL fluoxetine. Data represent the number of eggs released per animal after a 20-minute exposure to vehicle or drug. Error bars represent ±SEM from mean. *n* = 15 animals per condition, ***p < 0.001 ANOVA (Sidak). **(C-D)** Inactivation of *egl-2* via RNAi rescues *acox-1* egg-laying defects. Representative DIC images of day 1 adults of each genotype. **(C)** and quantification **(D)** of eggs retained in utero. Error bars indicate ±SEM from mean, *n* = 15 animals per genotype. ***p < 0.001 unpaired Student *t* test. See S1 Data for underlying data. *acox-1*, acyl-coenzyme A oxidase 1; DIC, differential interference contrast; *egl-2*, EGg Laying defective 2; RNAi, RNA interference.
(TIF)

S5 Fig Related to Fig 6. **(A)** RNAi-mediated knockdown of *egl-2* rescues egg-laying defects associated with aberrant channel activity *egl-2(n698)* mutants. Representative DIC images acquired from day 1 adults. DIC, differential interference contrast; *egl-2*, EGg Laying defective 2; RNAi, RNA interference.
(TIF)

S6 Fig Loss of *acox-1* suppresses URX body cavity neuron activity. **(A-D)** Activity of URX neurons in each indicated genotype using Ca^2+^ imaging by GCaMP5K under the control of the URX-specific *flp-8* promoter. Oxygen concentrations in the microfluidic chamber were 10% and 21%, as indicated. **(A-B)** For each genotype, black traces show the average percent change of GCaMP5K fluorescence (FΔ/F_0_) and gray shading indicates SEM. The number of animals used for each condition is shown in the figure. **(C-D)** Individual URX responses are shown for each genotype; each row represents one animal. **(E)** Maximal (FΔ/F_0_) values are shown for individual animals in wild-type and *acox-1* animals. Bars indicate the average value within each genotype. ***p < 0.001 by Student *t* test. **(F)** Individual baseline fluorescence (F_0_) values at 10% oxygen are shown for individual animals in wild-type and *acox-1* mutants. Bars indicate the median value within each genotype; n.s., not significant by Student *t* test. **(G)** We imaged mCherry fluorescence in wild-type and *acox*-1 mutant animals expressing both GCaMP5K and mCherry under the control of the *flp-8* promoter. Images were taken in animals exposed to 10% oxygen. **(H-I)** Activity of RIM neurons in wild-type and *acox-1* mutants using Ca^2+^ imaging by GCaMP3 under the control of the RIM specific *cex-1* promoter. **(H)** The total intensity of RIM GCaMP3 fluorescence in wild-type and *acox-1* mutants over a 250-second imaging window. **(I)** Maximal (FΔ/F_0_) values for wild-type and *acox-1* animals, *n* = 8 animals. See S1 Data for underlying data. *acox-1*, acyl-coenzyme A oxidase 1
(TIF)

S7 Fig Loss of *egl-2* does not rescue URX body cavity neuron activity in *acox-1* mutants. **(A-H)** Activity of URX neurons in each indicated genotype using Ca^2+^ imaging by GCaMP5K under the control of the URX specific *flp-8* promoter. Oxygen concentrations in the microfluidic chamber were 10% and 21%, as indicated. **(A-D)** For each genotype, black traces show the average percent change of GCaMP5K fluorescence (FΔ/F_0_) and gray shading indicates SEM. The number of animals used for each condition is shown in the figure. **(E-H)** Individual URX responses are shown for each genotype; each row represents one animal. **(I)** Maximal (FΔ/F_0_) values are shown for individual animals in wild-type, *acox-1*, *egl-2*, and *acox-1;egl-2* animals. Bars indicate the average value within each genotype. ***p < 0.001 by Student *t* test. **(J)** Individual baseline fluorescence (F_0_) values at 10% oxygen are shown for individual animals in wild-type, *acox-1*, *egl-2*, and *acox-1;egl-2* animal mutants. Bars indicate the median value within each genotype; n.s., not significant by Student *t* test. **(K)** We imaged mCherry fluorescence in wild-type and *acox*-1 mutant animals expressing both GCaMP5K and mCherry under the control of the *flp-8* promoter. Images were taken in animals exposed to 10% oxygen. See S1 Data for underlying data. *acox-1*, acyl-coenzyme A oxidase 1; *egl-2*, EGg Laying defective 2.
(TIF)

S8 Fig Intestine-specific reconstitution of *acox-1* does not rescue URX body cavity neuron activity in *acox-1* mutants. **(A-D)** Activity of URX neurons in each indicated genotype using Ca^2+^ imaging by GCaMP5K under the control of the URX-specific *flp-8* promoter. Oxygen concentrations in the microfluidic chamber were 10% and 21%, as indicated. **(A-B)** For each genotype, black traces show the average percent change of GCaMP5K fluorescence (FΔ/F_0_), and gray shading indicates SEM. The number of animals used for each condition is shown in the figure. **(C-D)** Individual URX responses are shown for each genotype; each row represents one animal. **(E)** Maximal (FΔ/F_0_) values are shown for individual animals in *acox-1* and *acox-1; vha-6p*::*(+)* (intestinal-specific rescue) animals. Bars indicate the average value within each genotype. ***p < 0.001 by Student *t* test. **(F)** Individual baseline fluorescence (F_0_) values at 10% oxygen are shown for individual animals in *acox-1* and *acox-1; vha-6p*::*(+)* mutants. Bars indicate the median value within each genotype; n.s., not significant by Student *t* test. **(G)** mCherry fluorescence in *acox-1* and *acox-1; vha-6p*::*(+)* mutant animals expressing both GCaMP5K and mCherry under the control of the *flp-8* promoter. Images were taken in animals exposed to 10% oxygen. See S1 Data for underlying data. *acox-1*, acyl-coenzyme A oxidase 1.
(TIF)

S1 Data Data underlying quantitative panels from Fig 1, Fig 2, Fig 3, Fig 5, Fig 6, Fig 7, S1 Fig, S2 Fig, S3 Fig, S4 Fig, S6 Fig, S7 Fig and S8 Fig. (XLSX)

S1 Table Unnormalized pharyngeal pumping data from Fig 1, Fig 3, Fig 6 and Fig 7. (XLSX)

S2 Table HPLC-ESI-MS data of differentially detected features in *acox-1* mutants. *acox-1*, acyl-coenzyme A oxidase 1; ESI, electrospray ionization; HPLC, high-pressure liquid chromatography; MS, mass spectrometry.
(XLSX)

S3 Table Sequences of primers used in this study. (XLSX)

## Abbreviations

AAK-2: AMP-activated kinase alpha 2
ACOX-1: acyl-coenzyme A oxidase 1
ACS: acyl-coenzyme A synthetase
AEA: arachidonoyl ethanolamide
AMPK: AMP-activated kinase
ascr#18: ascaroside #18
BAAT: bile acid-CoA: amino acid N-acyltransferase
CoA: co-enzyme A
DAGPE: diacylglycerophosphoethanolamine
DIC: differential interference contrast
EAG: *ether-a-go-go*
EGF: epidermal growth factor
ESI: electrospray ionization
FAAH-1: fatty acid amide hydrolase
GABA: Gamma-Aminobutyric Acid
gDNA: genomic DNA
GPI: glycosylphosphatidylinositol
HPLC: high-pressure liquid chromatography
HRMS: high-resolution mass spectrometry
MS/MS: tandem mass spectrometry
NAE: *N*-acylethanolamine
OEA: oleoyl ethanolamide
PDMS: poly(dimethylsiloxane)
PGAP2: post-glycophosphatidylinositol attachment to protein 2
PIP2: phosphatidylinositol 4,5-bisphosphate
PKC: protein kinase C
RNAi: RNA interference
ROI: region of interest
SPK: serine and arginine-rich protein kinase
5HT: 5-hydroxytryptamine
