# Supplementary figures and images for: Intestinal peroxisomal fatty acid β-oxidation regulates neural serotonin signaling through a feedback mechanism
Source: PLoS Biol. 2019 Dec 5;17(12):e3000242. doi: 10.1371/journal.pbio.3000242 (PMC6917301; doi:10.1371/journal.pbio.3000242)

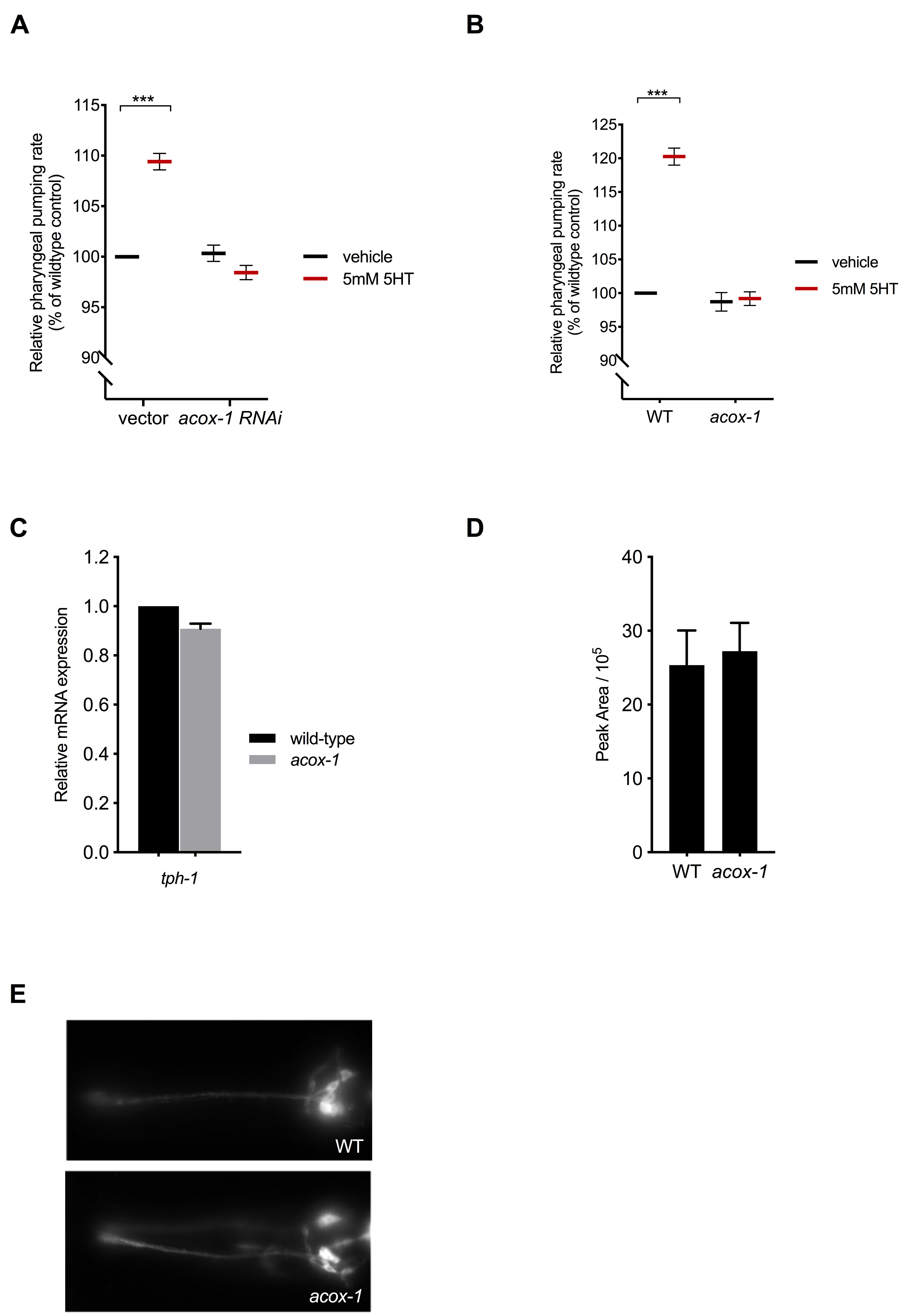

Supplement: S1 Fig — (A) Knocking down acox-1(F08A8.1) by RNAi suppresses the feeding elevating effects of exogenous serotonin (5 mM 5HT). Animals were treated with RNAi from the L1 stage, and feeding was assayed at day 1 adult stage. Feeding data are normalized to vehicle-treated wild-type animals and are presented as a percentage of wild-type animals. Error bars indicate ±SEM from normalized mean, n = 20 animals per strain. ***p < 0.001 ANOVA (Tukey). (B) Pharyngeal pumping rates were counted over a longer interval (60 seconds) from video recordings. Error bars indicate ±SEM from normalized mean, n = 10 animals per condition. ***p < 0.001 ANOVA (Tukey). See S1 Data for underlying data. (C) Loss of acox-1 does not influence the transcriptional expression of tryptophan hydroxylase (tph-1) as measured by qPCR. Error bars indicate ±SEM from mean n = 3 independent assays. (D) Relative abundance of 5HT in wild-type and acox-1 mutants, as determined by LC-HRMS. Error bars indicate ±SEM from mean, n = 4 independent experiments. (E) Loss of acox-1 does not grossly alter amphid neuron morphology. DiI staining of amphid chemosensory neurons in wild-type and acox-1 mutants. Images acquired at day 1 adult stage. acox-1, acyl-coenzyme A oxidase 1; HRMS, high-resolution mass spectrometry; LC, liquid chromatography; qPCR, quantitative PCR; RNAi, RNA interference; 5HT, 5-hydroxytryptamine. (TIF) [file pbio.3000242.s001.tif]

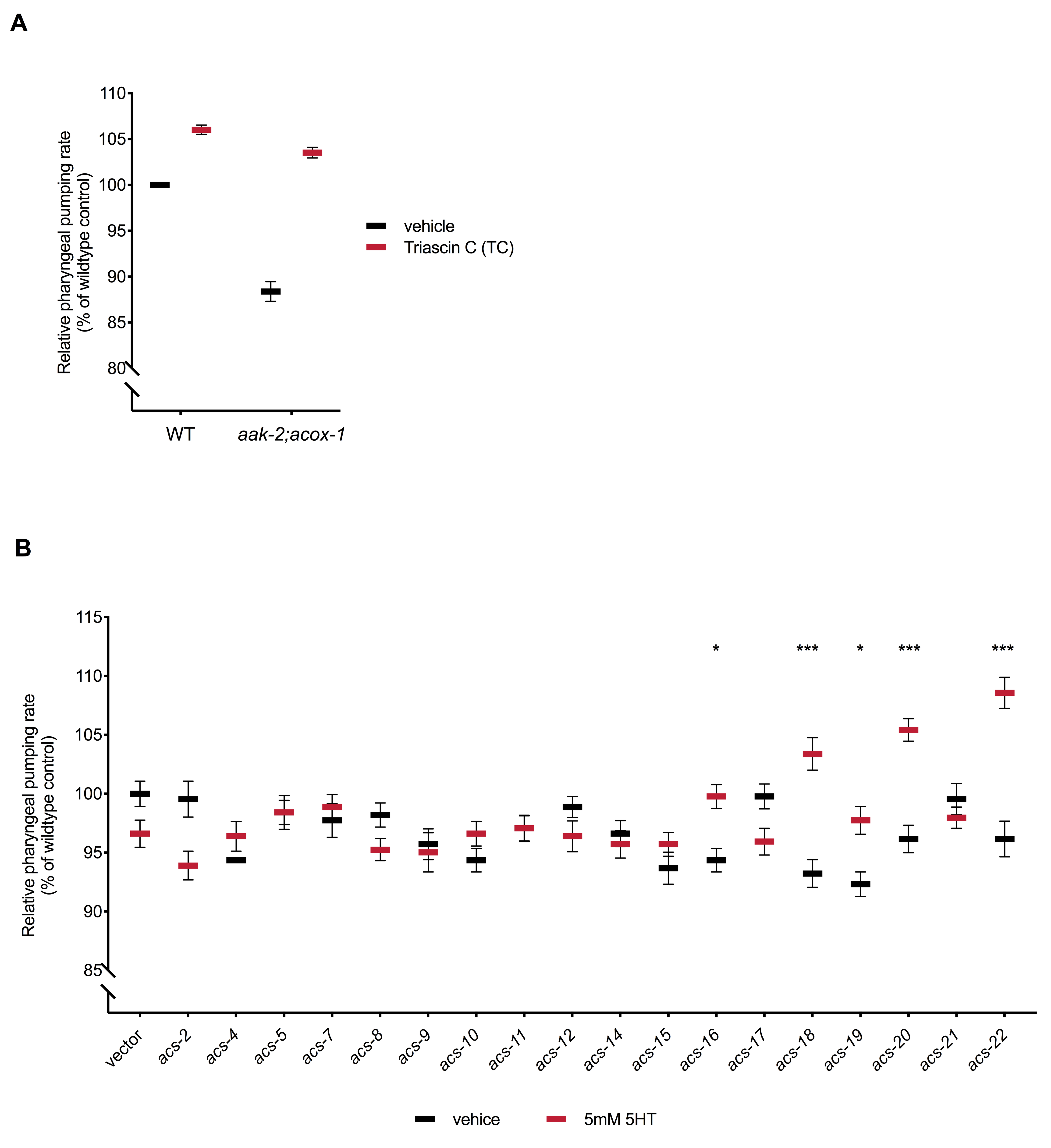

Supplement: S2 Fig — (A) Treating aak-2;acox-1 mutants with 1 μM triacsin C for 60 minutes elevates pharyngeal pumping rates. Day 1 adult animals were treated with to vehicle (0.0002% DMSO) or 1 μM triacsin C for 60 minutes before assessing pharyngeal pumping rates. Error bars indicate ±SEM from normalized mean, n = 15 animals per condition. At the concentration used, triacsin C had no effect on feeding rate. (B) RNAi-mediated inactivation of distinct ACSs suppress feeding defects in acox-1(ok2257) animals. Animals were treated with respective RNAi clones from L1 stage and feeding was assayed at day 1 adult stage. All feeding data are normalized to vehicle and vector RNAi-treated wild-type animals and are presented as a percentage of wild-type rates. Error bars indicate ±SEM from normalized mean, n = 15 animals per strain. *p < 0.05, ***p < 0.001 ANOVA (Tukey). See S1 Data for underlying data. CoA, co-enzyme A; RNAi, RNA interference. (TIF) [file pbio.3000242.s002.tif]

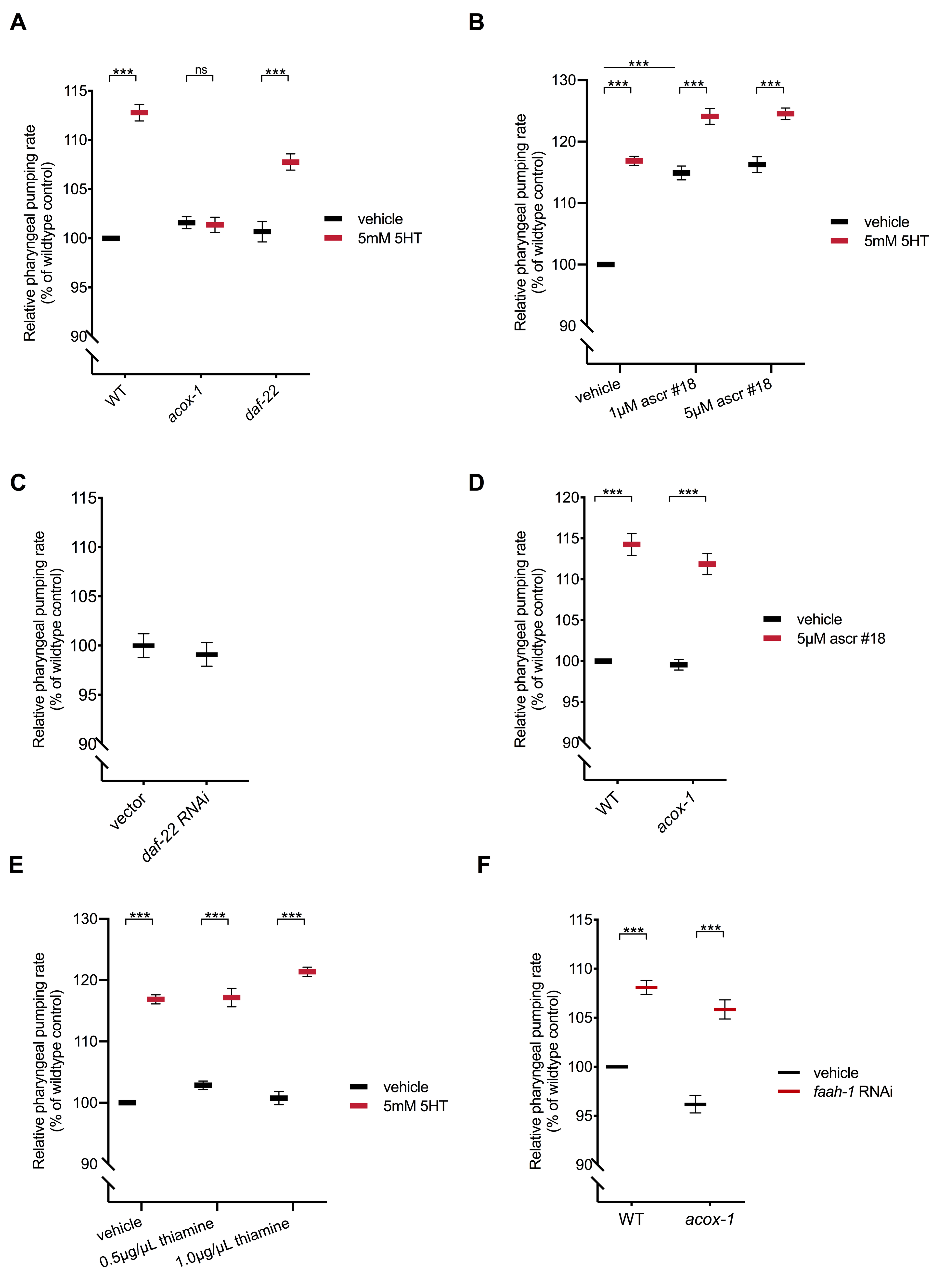

Supplement: S3 Fig — (A) daf-22(m130) animals are still responsive to the feeding elevating effects of 5 mM serotonin, n = 10 animals per condition. (B) Feeding responses of wild-type animals to ascr#18. Animals were exposed to 1 μM and 5 μM ascr#18 from the L1 stage, and pharyngeal pumping rates were determined at day 1 adult stage. n = 15 animals per condition. (C) Feeding responses of acox-1(ok2257) animals to vector and daf-22 RNAi, n = 15 animals per condition. (D) Feeding responses of wild-type and acox-1 mutants to 5 μM ascr#18, n = 15 animals per condition. (E) Feeding responses of wild-type animals to thiamine. Animals were exposed to 0.5 μg/μL thiamine and 1.0 μg/μL thiamine from L1 stage, and pharyngeal pumping rates were determined at day 1 adult stage. n = 15 animals per condition. (F) RNAi-mediated inactivation of FAAH-1 elevates feeding responses of wild-type and acox-1 mutants. Animals were grown on faah-1 RNAi from L1 stage and feeding was assayed at day 1 adult stage, n = 10 animals per condition. All feeding data are normalized to vehicle-treated wild-type animals and are presented as a percentage of wild-type animals. Error bars indicate ±SEM from normalized mean, ***p < 0.001 two-way ANOVA (Tukey). See S1 Data for underlying data. ascr#18, ascaroside #18; FAAH-1, fatty acid amide hydrolase; RNAi, RNA interference. (TIF) [file pbio.3000242.s003.tif]

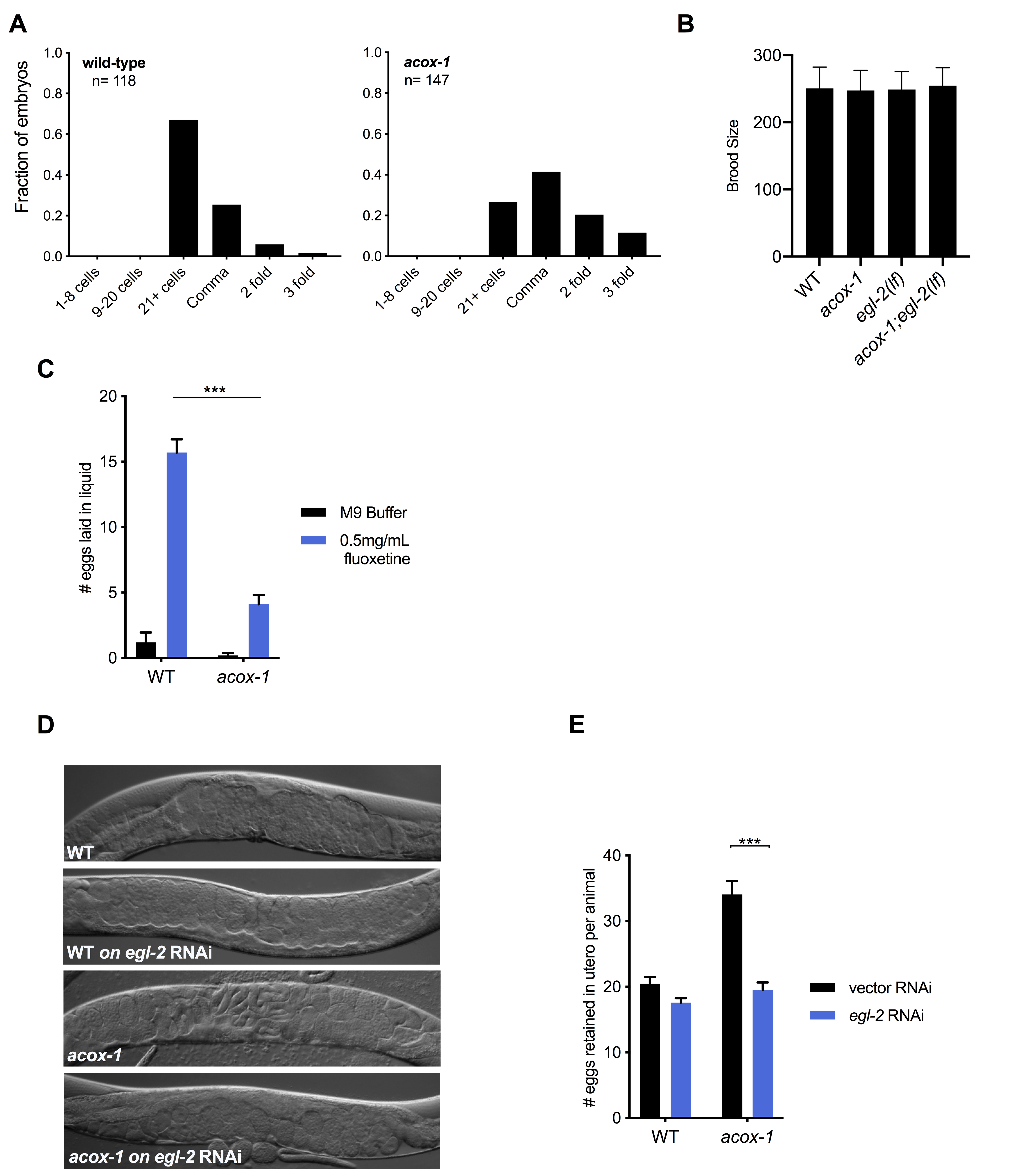

Supplement: S4 Fig — (A) acox-1(ok2257) mutants lay eggs at a later developmental stage than wild-type animals, suggesting that in utero retention time is increased. Histograms indicate the distribution of embryos at each developmental stage. (B) There was no significant difference in the total number of progeny between wild-type, acox-1, egl-2, and acox-1;egl-2 mutants. (C) acox-1 mutants are less responsive to the egg-laying inducing effects of serotonin. Egg-laying response of wild-type and acox-1 mutants in control buffer (M9) or 0.5 mg/mL fluoxetine. Data represent the number of eggs released per animal after a 20-minute exposure to vehicle or drug. Error bars represent ±SEM from mean. n = 15 animals per condition, ***p < 0.001 ANOVA (Sidak). (C-D) Inactivation of egl-2 via RNAi rescues acox-1 egg-laying defects. Representative DIC images of day 1 adults of each genotype. (C) and quantification (D) of eggs retained in utero. Error bars indicate ±SEM from mean, n = 15 animals per genotype. ***p < 0.001 unpaired Student t test. See S1 Data for underlying data. acox-1, acyl-coenzyme A oxidase 1; DIC, differential interference contrast; egl-2, EGg Laying defective 2; RNAi, RNA interference. (TIF) [file pbio.3000242.s004.tif]

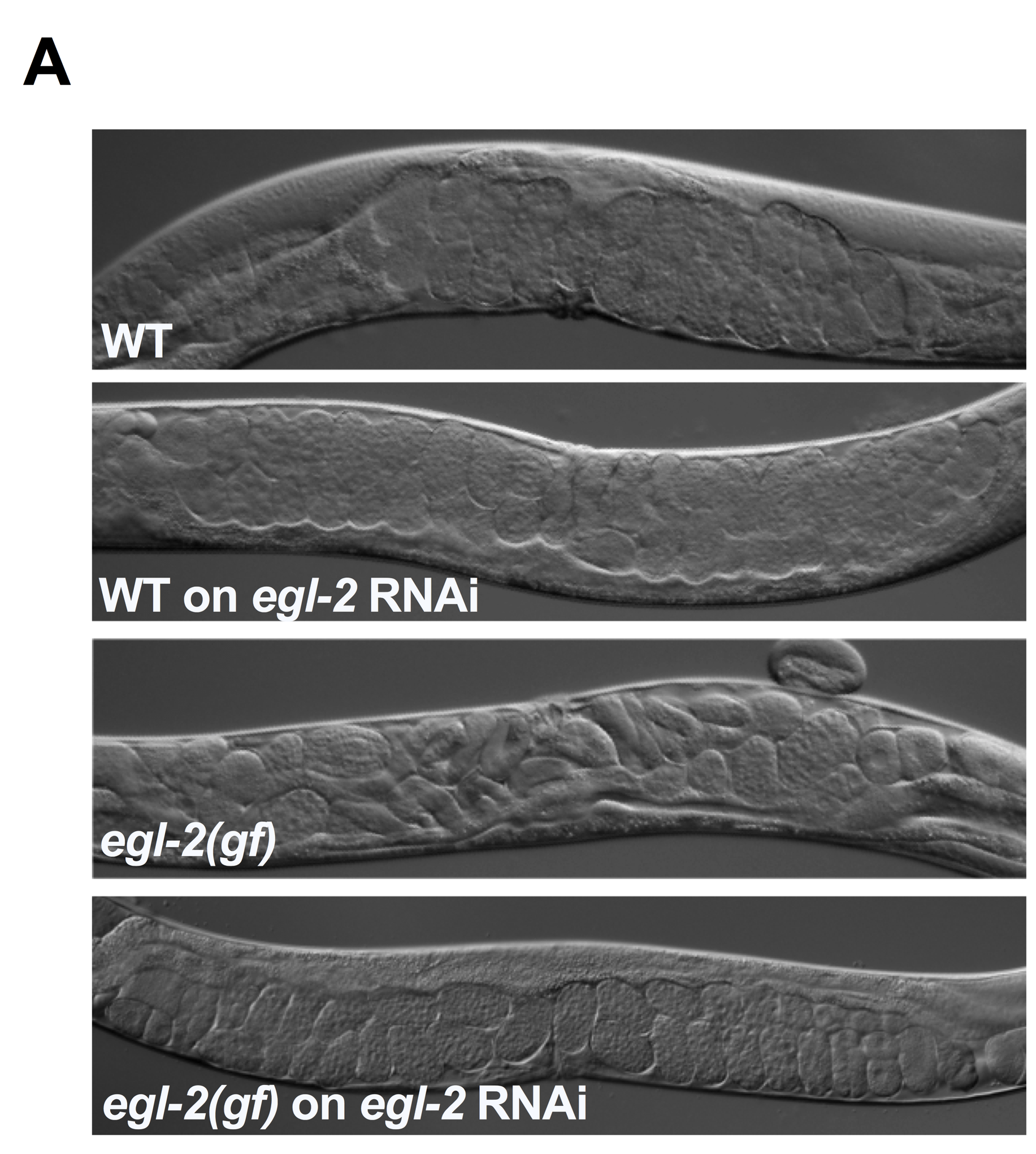

Supplement: S5 Fig — (A) RNAi-mediated knockdown of egl-2 rescues egg-laying defects associated with aberrant channel activity egl-2(n698) mutants. Representative DIC images acquired from day 1 adults. DIC, differential interference contrast; egl-2, EGg Laying defective 2; RNAi, RNA interference. (TIF) [file pbio.3000242.s005.tif]

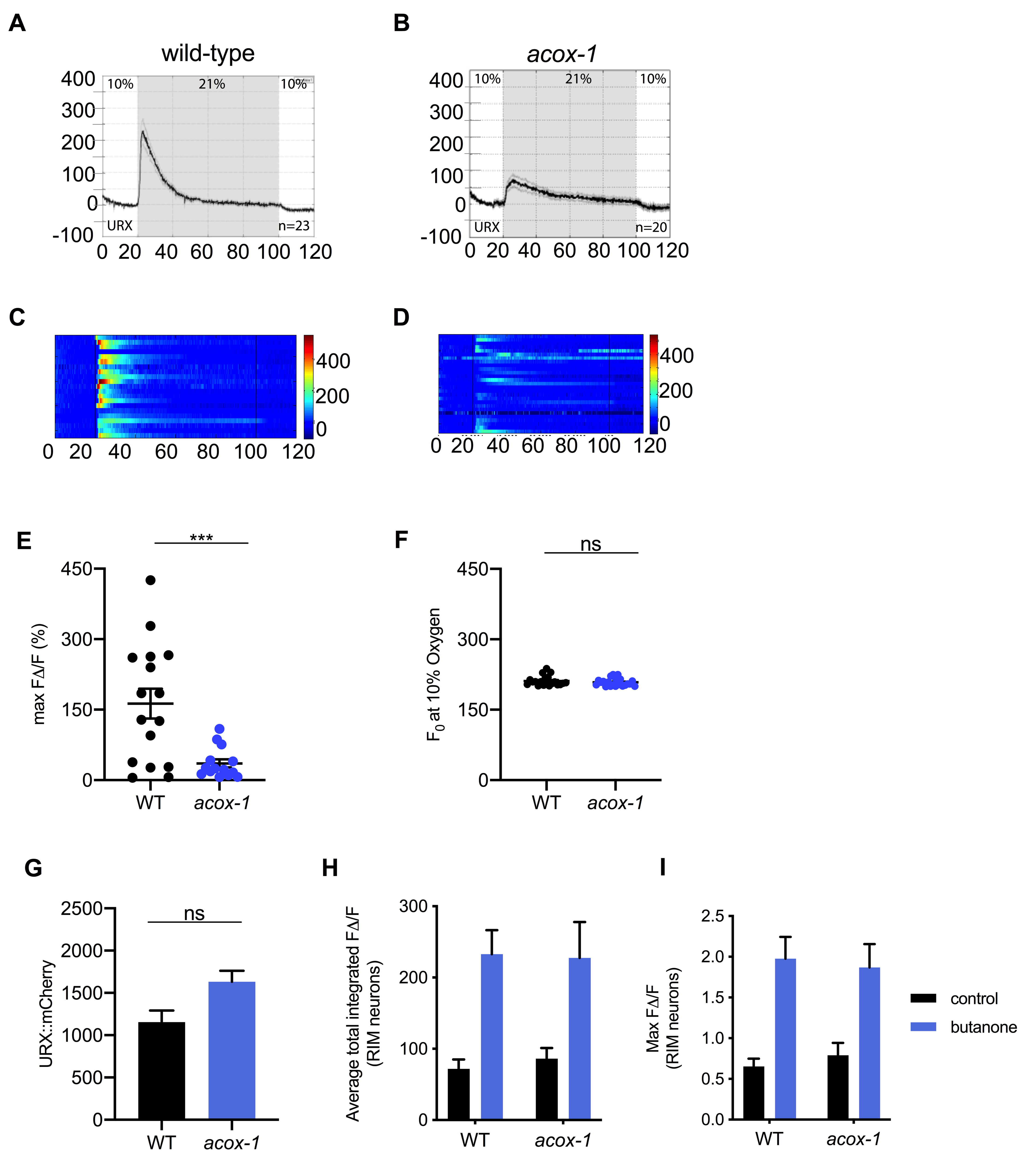

Supplement: S6 Fig — (A-D) Activity of URX neurons in each indicated genotype using Ca2+ imaging by GCaMP5K under the control of the URX-specific flp-8 promoter. Oxygen concentrations in the microfluidic chamber were 10% and 21%, as indicated. (A-B) For each genotype, black traces show the average percent change of GCaMP5K fluorescence (FΔ/F0) and gray shading indicates SEM. The number of animals used for each condition is shown in the figure. (C-D) Individual URX responses are shown for each genotype; each row represents one animal. (E) Maximal (FΔ/F0) values are shown for individual animals in wild-type and acox-1 animals. Bars indicate the average value within each genotype. ***p < 0.001 by Student t test. (F) Individual baseline fluorescence (F0) values at 10% oxygen are shown for individual animals in wild-type and acox-1 mutants. Bars indicate the median value within each genotype; n.s., not significant by Student t test. (G) We imaged mCherry fluorescence in wild-type and acox-1 mutant animals expressing both GCaMP5K and mCherry under the control of the flp-8 promoter. Images were taken in animals exposed to 10% oxygen. (H-I) Activity of RIM neurons in wild-type and acox-1 mutants using Ca2+ imaging by GCaMP3 under the control of the RIM specific cex-1 promoter. (H) The total intensity of RIM GCaMP3 fluorescence in wild-type and acox-1 mutants over a 250-second imaging window. (I) Maximal (FΔ/F0) values for wild-type and acox-1 animals, n = 8 animals. See S1 Data for underlying data. acox-1, acyl-coenzyme A oxidase 1 (TIF) [file pbio.3000242.s006.tif]

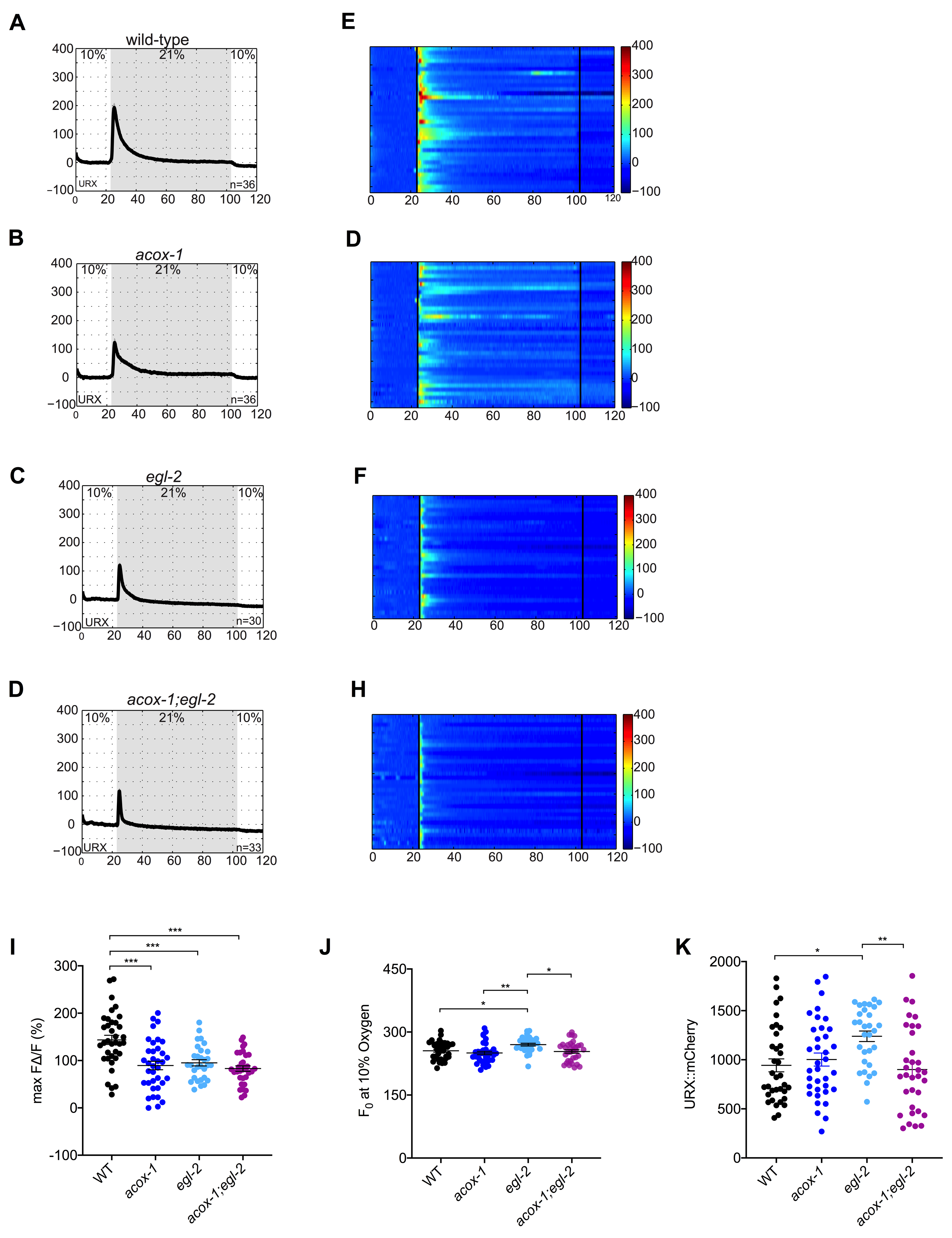

Supplement: S7 Fig — (A-H) Activity of URX neurons in each indicated genotype using Ca2+ imaging by GCaMP5K under the control of the URX specific flp-8 promoter. Oxygen concentrations in the microfluidic chamber were 10% and 21%, as indicated. (A-D) For each genotype, black traces show the average percent change of GCaMP5K fluorescence (FΔ/F0) and gray shading indicates SEM. The number of animals used for each condition is shown in the figure. (E-H) Individual URX responses are shown for each genotype; each row represents one animal. (I) Maximal (FΔ/F0) values are shown for individual animals in wild-type, acox-1, egl-2, and acox-1;egl-2 animals. Bars indicate the average value within each genotype. ***p < 0.001 by Student t test. (J) Individual baseline fluorescence (F0) values at 10% oxygen are shown for individual animals in wild-type, acox-1, egl-2, and acox-1;egl-2 animal mutants. Bars indicate the median value within each genotype; n.s., not significant by Student t test. (K) We imaged mCherry fluorescence in wild-type and acox-1 mutant animals expressing both GCaMP5K and mCherry under the control of the flp-8 promoter. Images were taken in animals exposed to 10% oxygen. See S1 Data for underlying data. acox-1, acyl-coenzyme A oxidase 1; egl-2, EGg Laying defective 2. (TIF) [file pbio.3000242.s007.tif]

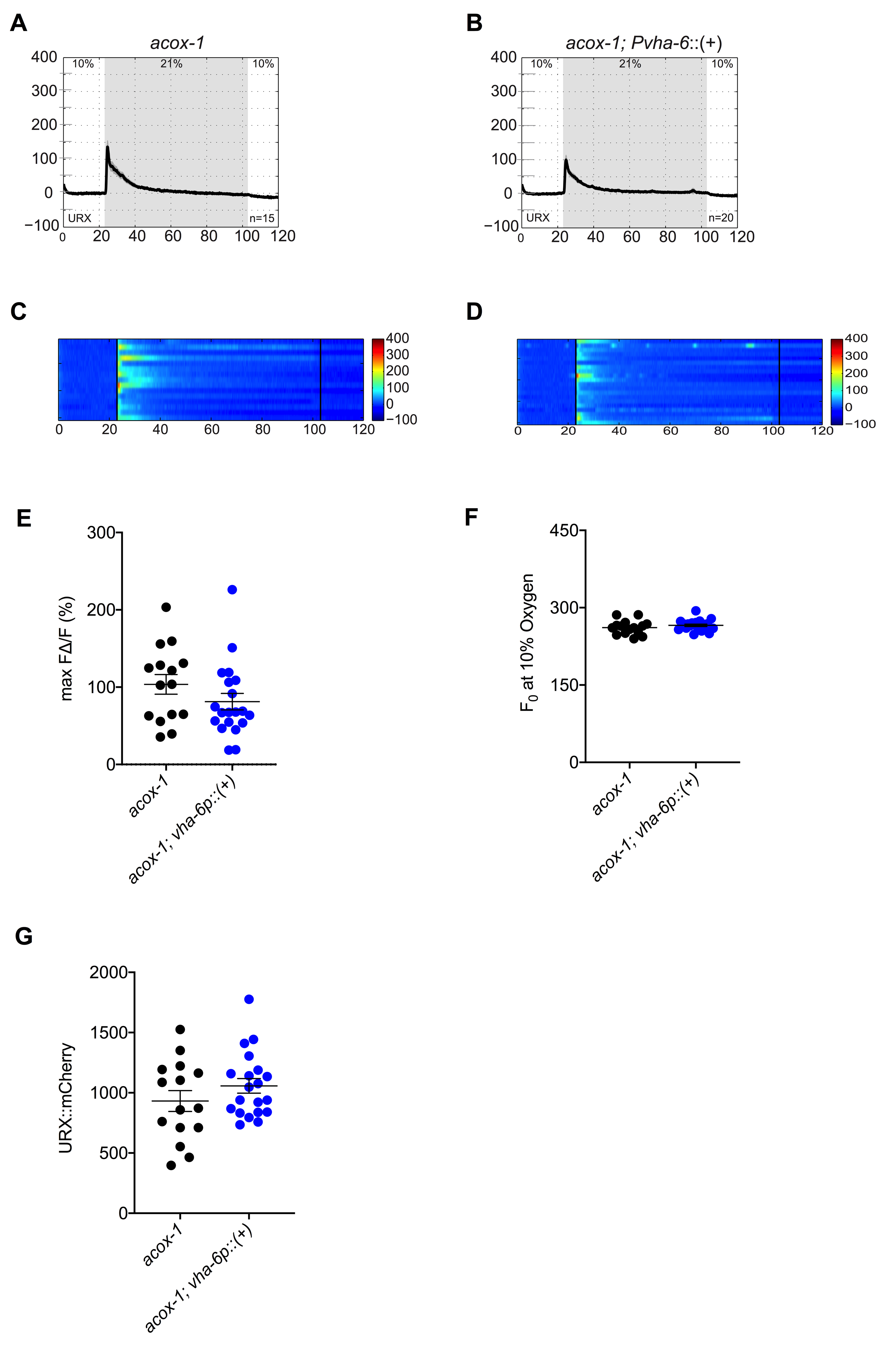

Supplement: S8 Fig — (A-D) Activity of URX neurons in each indicated genotype using Ca2+ imaging by GCaMP5K under the control of the URX-specific flp-8 promoter. Oxygen concentrations in the microfluidic chamber were 10% and 21%, as indicated. (A-B) For each genotype, black traces show the average percent change of GCaMP5K fluorescence (FΔ/F0), and gray shading indicates SEM. The number of animals used for each condition is shown in the figure. (C-D) Individual URX responses are shown for each genotype; each row represents one animal. (E) Maximal (FΔ/F0) values are shown for individual animals in acox-1 and acox-1; vha-6p::(+) (intestinal-specific rescue) animals. Bars indicate the average value within each genotype. ***p < 0.001 by Student t test. (F) Individual baseline fluorescence (F0) values at 10% oxygen are shown for individual animals in acox-1 and acox-1; vha-6p::(+) mutants. Bars indicate the median value within each genotype; n.s., not significant by Student t test. (G) mCherry fluorescence in acox-1 and acox-1; vha-6p::(+) mutant animals expressing both GCaMP5K and mCherry under the control of the flp-8 promoter. Images were taken in animals exposed to 10% oxygen. See S1 Data for underlying data. acox-1, acyl-coenzyme A oxidase 1. (TIF) [file pbio.3000242.s008.tif]
